# Distant metastasis risk and prognosis in elderly gastric cancer patients after neoadjuvant chemotherapy and surgery

**DOI:** 10.3389/fonc.2026.1757874

**Published:** 2026-02-06

**Authors:** Jiarong Shang, Jin Zhu, Xia Zheng, Yujia Shao, Jun Qian, Yong Li, Ping Wang

**Affiliations:** 1 Shanghai Municipal Hospital of Traditional Chinese Medicine, Shanghai University of Traditional Chinese Medicine, Shanghai, China; 2 Oncology Department, Jiangsu Province Hospital of Chinese Medicine, Affiliated Hospital of Nanjing University of Chinese Medicine, Nanjing, Jiangsu, China; 3 Department of Integrated Traditional Chinese and Western Medicine, The Third Affiliated Hospital of Bengbu Medical University, Bengbu, China; 4 Emergency & Critical Care Center, Jiangsu Province Hospital of Chinese Medicine, Affiliated Hospital of Nanjing University of Chinese Medicine, Nanjing, Jiangsu, China

**Keywords:** gastric cancer, neoadjuvant chemotherapy, distant metastasis, elderly, nomogram, cox regression, survival

## Abstract

**Background:**

Gastric cancer imposes a heavy global health burden, and treatment evaluation in elderly patients is often more complex. Although NAC is standard for locally advanced gastric cancer (LAGC), benefits in the elderly are heterogeneous, postoperative distant metastasis (DM) is underexplored, and no nomogram specifically evaluates postoperative DM diagnosis and prognosis in elderly LAGC after NAC.

**Methods:**

This study extracted data from patients over 70 years of age who were diagnosed with gastric adenocarcinoma and underwent neoadjuvant chemotherapy followed by curative gastrectomy between 2016 and 2022. Independent risk factors for postoperative distant metastasis following neoadjuvant chemotherapy were identified using univariate and multivariate logistic regression analyses, while independent prognostic factors were determined through univariate and multivariate Cox proportional hazards regression analyses. Subsequently, we developed two novel nomograms and evaluated their performance using receiver operating characteristic (ROC) curves, calibration curves, and decision curve analysis (DCA).

**Results:**

A total of 896 elderly gastric adenocarcinoma patients were enrolled, among whom 307 (34.26%) developed postoperative DM. Independent risk factors for DM included N stage, NAC-related adverse events, CA19–9 levels, NLR, tumor nodules, resection margin status, tumor regression grade, as well as intraoperative and postoperative chemotherapy. Among DM patients, independent prognostic predictors included CA72–4 levels, NLR, NAC-to-surgery interval, tumor regression grade, resection margin status, and postoperative chemotherapy. Both nomograms demonstrated high predictive accuracy, supported by ROC analysis, calibration curves, decision curve analysis, and Kaplan-Meier survival analysis in the training and validation sets.

**Conclusions:**

The two nomograms show promise as effective tools for predicting the risk of postoperative distant metastasis and estimating personalized prognosis in elderly gastric cancer patients following neoadjuvant chemotherapy, thereby potentially informing clinical decision-making.

## 1 Introduction

Gastric cancer (GC) remains a major global public health burden, ranking as the fifth most commonly diagnosed malignancy and the fourth leading cause of cancer-related mortality worldwide. Approximately one million new cases are diagnosed each year, resulting in more than 650,000 deaths (1). With the accelerating trend of population aging, there has been a continuous increase in the number of elderly gastric cancer patients and their demand for diagnostic and therapeutic services. Epidemiological studies indicate that the median age at diagnosis of gastric cancer exceeds 70 years, and the aging population is projected to be the primary driver of rising incidence and mortality (2–4). Compared with younger patients, elderly patients more frequently present with multimorbidity, frailty, and malnutrition, resulting in a more complex risk-benefit profile for both surgical and systemic therapies. This underscores the urgent need for precise stratification and individualized management.

Neoadjuvant chemotherapy (NAC) has become a cornerstone perioperative strategy for locally advanced gastric cancer (LAGC), demonstrating the potential to improve R0 resection rates, achieve tumor downstaging, and confer survival benefits (5). Recent studies also suggest that NAC can improve oncological outcomes and long-term survival in elderly LAGC patients (6, 7). However, significant heterogeneity exists among elderly patients in terms of tolerance to NAC and the actual survival benefit derived from it. Previous research has predominantly focused on overall survival (OS) or pathological response as primary endpoints, with less systematic characterization of the risk of postoperative distant metastasis (DM) as a key outcome. In reality, DM is a critical event in gastric cancer mortality. Once it occurs, patient OS is significantly shortened, treatment strategies often shift to palliative intent, and clinical outcomes deteriorate rapidly. Epidemiological data indicate that approximately one-third of gastric cancer patients present with distant metastasis at initial diagnosis, with a dismal 5-year relative survival rate of only about 7%–8% and a median overall survival typically under one year (8). Common metastatic sites in gastric cancer include the liver, peritoneum, lungs, bones, and distant lymph nodes. Among patients with metastatic disease, liver involvement is reported in approximately 43%–48% of cases, peritoneal involvement in about 32%, pulmonary involvement in about 15%, and bone involvement in about 12%. Prognosis varies considerably depending on the metastatic site. Several recent studies indicate that median overall survival for peritoneal metastasis is typically only a few months to within a year, while median OS for liver metastasis is often approximately 10–12 months, although outcomes are significantly influenced by treatment modalities and metastatic burden (9–12). Therefore, identifying high-risk subgroups for DM among elderly gastric cancer patients who have undergone NAC and subsequent radical resection, and implementing personalized follow-up and intervention strategies at an early stage, represents a clear clinical necessity.

The nomogram has been widely adopted in recent years to evaluate cancer prognosis due to its convenience and precision, making it a suitable tool for our purpose (13). Accordingly, we conducted a retrospective study enrolling a consecutive cohort of elderly gastric cancer patients who underwent radical resection following NAC. This study aimed to determine the incidence, risk factors, and prognosis of postoperative distant metastasis in this population, and to develop two nomograms: one to predict the risk of postoperative DM, and another to predict OS among those who developed DM after NAC and radical surgery.

## 2 Patients and methods

### 2.1 Patients

The data for this study were derived from patients with locally advanced gastric cancer who underwent neoadjuvant chemotherapy followed by radical gastrectomy at Jiangsu Provincial Hospital of Traditional Chinese Medicine between October 2016 and December 2022. The study protocol was approved by the Institutional Review Board of Jiangsu Provincial Hospital of Traditional Chinese Medicine, affiliated with Nanjing University of Chinese Medicine (Approval No. 2022NL-137-01). All procedures were conducted in accordance with the ethical standards of the institutional research committee and with the Helsinki Declaration. Inclusion criteria were: (1) age ≥70 years; (2) preoperative pathological confirmation of primary gastric adenocarcinoma; (3) clinical stage cT1-2N1-3M0 or cT3-4N0-3M0; (4) receipt of NAC followed by radical gastrectomy; and (5) availability of complete clinicopathological data. Exclusion criteria were: (1) unsuitability for radical resection; (2) history of other malignant tumors; (3) preoperative radiotherapy; (4) any neoadjuvant treatment other than chemotherapy; (5) incomplete clinicopathological data; (6) metastasis or death within 6 months after surgery; or (7) loss to follow-up. Based on these criteria, a final cohort of 896 elderly LAGC patients was enrolled, among whom 307 developed distant metastasis (DM). The entire cohort served as the diagnostic cohort for identifying risk factors for DM and developing a predictive nomogram. The 307 patients who developed DM constituted the prognostic cohort, which was used to investigate prognostic factors and construct a prognostic nomogram for this subgroup. In both cohorts, patients were randomly allocated to a training set (70%) and a validation set (30%) at a 7:3 ratio. For each cohort, the nomogram was developed using the training set and subsequently validated using the validation set.

### 2.2 Data collection

In this study, the following variables were selected to identify risk factors for distant metastasis in elderly patients with locally advanced gastric adenocarcinoma who underwent radical gastrectomy after neoadjuvant chemotherapy: (1) Demographics and Comorbidities: Age, sex, marital status, body mass index (BMI, kg/m²); comorbidities included diabetes mellitus, cerebral infarction, coronary heart disease, and hypertension.

(2) Neoadjuvant Treatment and Toxicity: NAC regimen, cycle number, grade ≥3 adverse events (per CTCAE criteria), and the interval from NAC completion to surgery; (3) Surgery and Perioperative Details: Surgical extent, operative approach, intraoperative blood loss (mL), operative duration (minutes), and whether intraoperative chemotherapy was administered. (4) Postoperative Complications: Complications were graded according to the Clavien-Dindo classification, with a grade ≥II defined as a positive event (a sensitivity analysis using grade ≥III was also planned). (5) Preoperative Laboratory and Tumor Markers: Hemoglobin (g/L), white blood cell count, absolute neutrophil count, lymphocyte count, platelet count (all ×10^9^/L), and albumin (g/L); carcinoembryonic antigen (CEA), CA19-9, CA125, and CA72-4. The following ratios were calculated from the same blood test: neutrophil-to-lymphocyte ratio (NLR), platelet-to-lymphocyte ratio (PLR), and systemic immune-inflammation index (SII = platelets × neutrophils/lymphocytes). (6) Postoperative Pathology and Staging: presence of signet-ring cell component, differentiation grade, Lauren classification, resection margin status, pathological T and N stage (AJCC 8th edition), and presence of vascular invasion and perineural invasion. Pathological response to NAC was assessed using the tumor regression grade (TRG) system (14): Grade 0 (complete regression), Grade 1 (significant regression), Grade 2 (partial regression), Grade 3 (no evident regression). TRG 0–2 was defined as NAC benefit, and TRG 3 as no benefit. (7) Adjuvant Treatment: Receipt of postoperative chemotherapy and the number of cycles administered. (8) Outcomes and Follow-up: The primary outcome was the occurrence and time to the first DM after surgery. The site of the first DM (liver, lung, bone, brain, peritoneum, abdominal lymph nodes, cervical lymph nodes, other) was recorded. The secondary outcome was OS, calculated from the date of surgery. Follow-up was conducted via outpatient visits or telephone interviews until June 2025.

### 2.3 Statistical analysis

**Figure 1:**
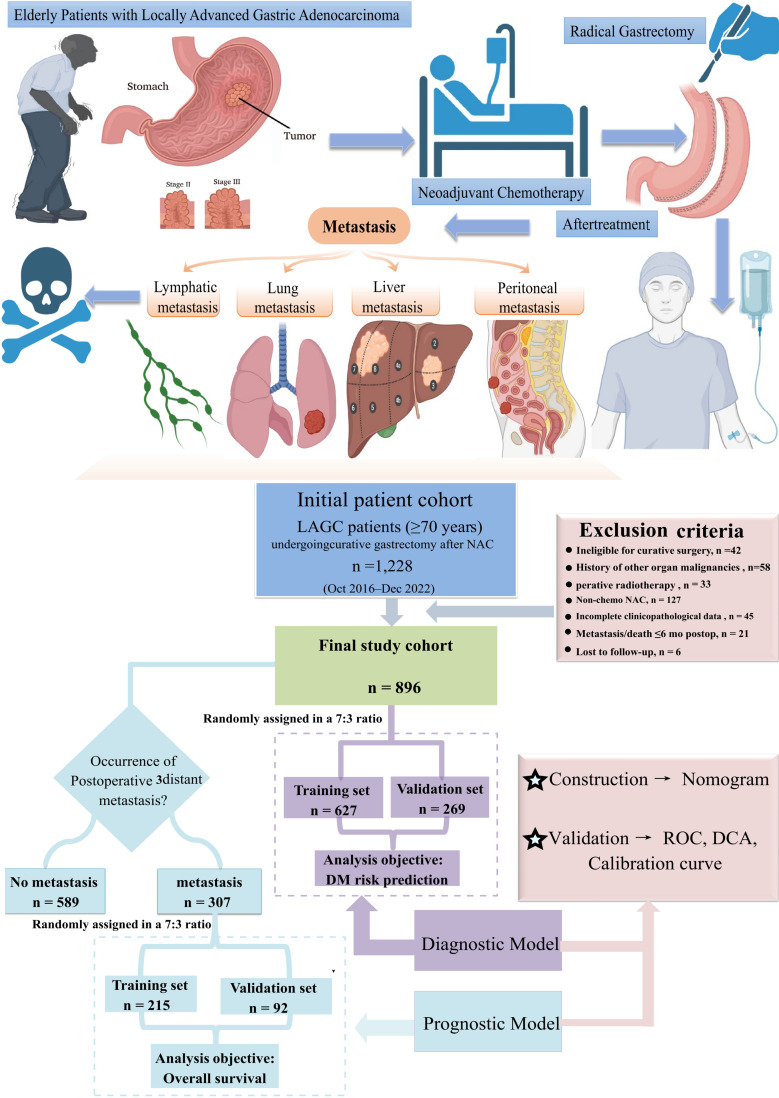
Study flowchart and model construction for elderly patients with LAGC.

All statistical analyses in this study were performed using SPSS (version 24.0) and R software (version 4.3.2). A two-sided P-value of less than 0.05 was considered statistically significant. The entire patient cohort was randomly split into a training set and a validation set in a 7:3 ratio. Categorical variables were compared between the two sets using the Chi-square test or Fisher’s exact test, as appropriate. Continuous variables were assessed for normality and homogeneity of variance; accordingly, the Student’s t-test or the Mann-Whitney U test was applied for group comparisons. Prior to model construction, restricted cubic splines (RCS) were used to evaluate the functional form of continuous predictors. For logistic regression, predictors were assessed against the log-odds, and for Cox regression, against the log-hazard. In exploratory analyses, RCS were fitted with three knots placed at recommended percentiles (10th, 50th, and 90th). When the relationship appeared approximately linear, the variable was entered into the final model as a linear term. If evident non-linearity was observed, and a reliable threshold was identified in the training cohort using X-tile, the variable was categorized accordingly. For the diagnostic cohort, univariate logistic regression analyses were first performed to identify variables associated with DM. Variables with a P-value < 0.05 in the univariate analysis were subsequently included in a multivariate logistic regression model to identify independent risk factors. Based on these independent factors, a diagnostic nomogram was constructed using the rms package in R. The model’s discriminatory ability was evaluated using Receiver Operating Characteristic (ROC) curves and the corresponding Area Under the Curve (AUC). Calibration curves and Decision Curve Analysis (DCA) were used to assess calibration and clinical utility, respectively. For the prognostic cohort (patients who developed DM), univariate Cox regression analyses were conducted to identify factors associated with OS. Significant variables (P < 0.05) were then entered into a multivariate Cox proportional hazards regression analysis using the “Forward LR” method to determine independent prognostic factors. A prognostic nomogram was developed based on these factors. Time-dependent ROC curves at 24, 36, and 60 months and time-dependent AUC values were generated to evaluate discriminatory ability over time. Calibration and DCA were also performed at these time points. Based on the median risk score derived from the nomogram, patients were categorized into high- and low-risk groups. Kaplan-Meier survival curves were plotted, and the log-rank test was used to compare OS between the groups. The overall study workflow is summarized in **Figure 1**.

## 3 Results

### 3.1 Baseline characteristics of the study population

A total of 896 elderly patients with locally advanced gastric adenocarcinoma were enrolled in the study and randomly assigned to a training set (n = 627) and a validation set (n = 269). The mean age was 75.2 ± 4.31 years in the training set and 75.1 ± 4.51 years in the validation set. As shown in **Table 1**, the distribution of tumor stages was comparable between the two sets. The T3 stage was the most prevalent (46.9% in the training set and 42.8% in the validation set), and the N0 nodal stage was predominant (33.5% and 37.2%, respectively). Regarding neoadjuvant chemotherapy, the SOX regimen was the most frequently used (44.3% in the training set vs. 40.1% in the validation set). The mean number of NAC cycles was approximately 3 in both groups, and the incidence of any grade ≥3 chemotherapy-related adverse event was approximately 20%–23%. Open surgery was the primary operative approach (83.3% vs. 87.0%), with R0 resection rates of 88.0% in the training set and 91.1% in the validation set. The incidence of postoperative complications was 31.3% and 26.4% in the training and validation sets, respectively. Pathological examination revealed that poorly differentiated/undifferentiated tumors accounted for approximately 60% of cases, while a signet-ring cell component was observed in about 16%–18%. The distribution of tumor regression grade was similar between the two sets. Overall, the baseline characteristics were well-balanced between the training and validation sets. No statistically significant differences were observed in any of the compared variables (p > 0.05, Chi-square or Fisher’s exact test), as detailed in **Table 1**. The linearity assumption of continuous variables with the logit of the outcome was tested using restricted cubic splines (RCS) (see **Supplementary Table S1**). The results indicated that apart from the Neutrophil-to-Lymphocyte Ratio (NLR), all other continuous variables approximately met the linearity assumption and were therefore included as linear terms in the model. NLR demonstrated a significant nonlinear relationship. The optimal cutoff value for NLR, determined using X-tile software, was 2.2, and it was subsequently categorized accordingly for analysis.

**Table 1:** Baseline clinical characteristics of elderly patients with locally advanced gastric cancer.

Variable	Training (N = 627)	Validation (N = 269)	χ²/Z	P
Sex			1.075	0.300
Male	470 (75.0%)	192 (71.4%)		
Female	157 (25.0%)	77 (28.6%)		
Age, years	75.2 (4.31)	75.1 (4.51)	0.308	0.758
T stage			3.155	0.532
T1	96 (15.3%)	50 (18.6%)		
T2	93 (14.8%)	42 (15.6%)		
T3	294 (46.9%)	115 (42.8%)		
T4	144 (23.0%)	62 (23.0%)		
N stage			2.959	0.398
N0	210 (33.5%)	100 (37.2%)		
N1	105 (16.7%)	39 (14.5%)		
N2	150 (23.9%)	71 (26.4%)		
N3	162 (25.8%)	59 (21.9%)		
Tumor location			3.421	0.490
Antrum	216 (34.4%)	106 (39.4%)		
Cardia	228 (36.4%)	99 (36.8%)		
Corpus	149 (23.8%)	51 (19.0%)		
Fundus	17 (2.7%)	6 (2.2%)		
Pylorus	17 (2.7%)	7 (2.6%)		
NAC regimen			3.376	0.642
FLOT	74 (11.8%)	30 (11.2%)		
FOLFOX	45 (7.2%)	26 (9.7%)		
Oral	77 (12.3%)	32 (11.9%)		
SOX	278 (44.3%)	108 (40.1%)		
XELOX	122 (19.5%)	61 (22.7%)		
Other	31 (4.9%)	12 (4.5%)		
NAC cycles, n	3.17 (0.754)	3.14 (0.756)	0.545	0.586
Diabetes			0.310	0.577
No	515 (82.1%)	216 (80.3%)		
Yes	112 (17.9%)	53 (19.7%)		
Stroke			0.001	0.974
No	522 (83.3%)	223 (82.9%)		
Yes	105 (16.7%)	46 (17.1%)		
CAD			1.074	0.300
No	562 (89.6%)	234 (87.0%)		
Yes	65 (10.4%)	35 (13.0%)		
Hypertension			0.697	0.404
No	343 (54.7%)	156 (58.0%)		
Yes	284 (45.3%)	113 (42.0%)		
BMI, kg/m²	23.3 (3.40)	22.9 (3.34)	1.63	0.103
Marital status			0.481	0.923
Married	332 (53.0%)	140 (52.0%)		
Single/Divorced	280 (44.6%)	124 (46.1%)		
Unknown	15 (2.4%)	5 (1.9%)		
NAC adverse events			0.668	0.414
No	484 (77.2%)	215 (79.9%)		
Yes	143 (22.8%)	54 (20.1%)		
CEA, ug/L			0.644	0.422
<5	445 (71.0%)	183 (68.0%)		
≥5	182 (29.0%)	86 (32.0%)		
CA19-9, U/mL			1.963	0.161
<37	500 (79.7%)	226 (84.0%)		
≥37	127 (20.3%)	43 (16.0%)		
CA125, U/mL			0.486	0.627
<35	449 (71.6%)	197 (73.2%)		
≥35	178 (28.4%)	72 (26.8%)		
CA72-4, U/mL			1.183	0.237
<7	437 (69.7%)	183 (68.0%)		
>7	190 (30.3%)	86 (32.0%)		
Intraoperative chemotherapy			0.429	0.512
No	380 (60.6%)	170 (63.2%)		
Yes	247 (39.4%)	99 (36.8%)		
Hemoglobin, g/L	111 (23.0)	111 (23.3)	0.000	1.000
WBC, ×10^9^/L	8.61 (3.12)	8.57 (3.20)	0.173	0.863
Neutrophils, ×10^9^/L	4.82 (3.03)	4.81 (3.04)	0.045	0.964
Platelets, ×10^9^/L	194 (70.3)	188 (70.4)	1.168	0.243
Albumin, g/L	36.6 (4.75)	36.7 (4.33)	0.308	0.758
Lymphocytes, ×10^9^/L	2.12 (0.624)	2.11 (0.640)	0.216	0.829
PLR	94.0 (35.4)	91.2 (34.6)	1.101	0.271
NLR	2.17 (0.748)	2.18 (0.732)	0.187	0.852
SII	425 (234)	413 (222)	0.729	0.466
Interval to surgery, days	43.6 (8.08)	43.7 (7.91)	0.964	0.669
Surgical approach			0.173	0.863
Laparoscopic	105 (16.7%)	35 (13.0%)		
Open	522 (83.3%)	234 (87.0%)		
Gastrectomy extent			1.374	0.503
Proximal	147 (23.4%)	61 (22.7%)		
Total	242 (38.6%)	95 (35.3%)		
Distal	238 (38.0%)	113 (42.0%)		
Blood loss, mL	179 (142)	177 (104)	0.235	0.814
Operative time, min	151 (42.4)	147 (43.2)	1.276	0.202
Postoperative complications			1.904	0.168
No	431 (68.7%)	198 (73.6%)		
Yes	196 (31.3%)	71 (26.4%)		
Vascular tumor thrombus			0.191	0.662
No	331 (52.8%)	147 (54.6%)		
Yes	296 (47.2%)	122 (45.4%)		
Perineural invasion			0.252	0.616
No	358 (57.1%)	148 (55.0%)		
Yes	269 (42.9%)	121 (45.0%)		
Tumor nodules			0.011	0.915
No	528 (84.2%)	228 (84.8%)		
Yes	99 (15.8%)	41 (15.2%)		
Lauren			0.222	0.147
Unknown	175 (27.9%)	63 (23.4%)		
Mixed	189 (30.1%)	98 (36.4%)		
Diffuse	150 (23.9%)	69 (25.7%)		
Intestinal	113 (18.0%)	39 (14.5%)		
Signet-ring cell component				
No	517 (82.5%)	226 (84.0%)		
Yes	110 (17.5%)	43 (16.0%)		
TRG			5.646	0.130
0	170 (27.1%)	73 (27.1%)		
1	241 (38.4%)	95 (35.3%)		
2	151 (24.1%)	82 (30.5%)		
3	65 (10.4%)	19 (7.1%)		
Histologic grade			0.121	0.728
Undifferentiated/Poor	387 (61.7%)	162 (60.2%)		
Well/Moderate	240 (38.3%)	107 (39.8%)		
Adjuvant chemotherapy			2.086	0.149
No	294 (46.9%)	141 (52.4%)		
Yes	333 (53.1%)	128 (47.6%)		
R status			2.341	0.310
R0	552 (88.0%)	245 (91.1%)		
R1	75 (12.0%)	24 (8.9%)		

### 3.2 Incidence and risk factors of postoperative distant metastasis

A total of 307 patients (34.26%) were confirmed to have developed distant metastasis, while 589 patients (65.74%) did not. Univariate logistic regression analysis was performed on 43 potential variables. Variables showing significant association (p < 0.05) were subsequently included in a multivariate logistic regression model (Results presented in **Table 2**; non-significant univariate results are listed in **Supplementary Table S2**). The multivariate logistic regression analysis identified the following as independent risk factors for postoperative DM in elderly patients with locally advanced gastric adenocarcinoma: a higher N stage, the occurrence of any NAC-related adverse event, elevated levels of CA19-9, an elevated neutrophil-to-lymphocyte ratio (NLR), the presence of tumor nodules, positive resection margins (R1), and a higher tumor regression grade (1/2/3 compared to 0). Conversely, intraoperative chemotherapy and postoperative adjuvant chemotherapy were identified as independent protective factors (**Table 2**).

**Table 2:** Univariate and multivariate logistic analyses of distant metastasis in elderly patients with locally advanced gastric cancer.

Variable (comparison)	Univariate analysis			Multivariate analysis		
	OR	95% CI	P	OR	95% CI	P
N stage (N1 vs N0)	1.753	1.022-2.994	0.04	1.642	0.780-3.439	0.189
N stage (N2 vs N0)	2.821	1.787-4.497	<0.001	2.062	1.010-4.236	0.047
N stage (N3 vs N0)	3.895	2.458-6.246	<0.001	3.184	1.662-6.188	<0.001
NAC adverse events (Yes vs No)	1.594	1.085-2.335	0.017	1.783	1.058-3.011	0.030
CA19-9(≥37 vs<37)	7.280	4.770-11.360	<0.001	1.014	1.003-1.026	0.017
CA125 (≥35 vs<35)	3.138	2.19-4.512	<0.001	0.826	0.455-1.475	0.524
CA72-4(≥7 vs<7)	2.175	1.530-3.096	<0.001	0.826	0.455-1.475	0.524
Intraoperative chemotherapy (Yes vs No)	0.381	0.263-0.545	<0.001	0.398	0.240-0.650	<0.001
Albumin (per unit)	0.944	0.910-0.979	0.002	0.969	0.921-1.016	0.201
NLR (per unit)	4.667	3.290-6.671	<0.001	4.261	(2.750-6.684)	<0.001
SII (per unit)	1.002	1.001-1.003	<0.001	1.000	0.999-1.002	0.69
Vascular tumor thrombus (Yes vs No)	1.743	1.251-2.436	0.001	0.787	0.447-1.374	0.402
Perineural invasion (Yes vs No)	1.983	1.421-2.775	<0.001	1.446	0.849-2.467	0.175
Tumor nodules (Yes vs No)	3.343	2.157-5.224	<0.001	2.356	1.278-4.377	0.006
Signet-ring component (Yes vs No)	2.132	1.403-3.240	<0.001	1.337	0.739-2.412	0.334
TRG (1 vs 0)	1.45	0.942-2.251	0.094	1.947	1.074-3.590	0.030
TRG (2 vs 0)	1.794	1.296-2.109	0.009	1.982	1.040-3.821	0.039
TRG (3 vs 0)	5.597	3.051-10.536	<0.001	7.928	3.559-18.281	<0.001
Histologic grade (Well/Moderate vs Poor/Undiff.)	0.516	0.360-0.732	<0.001	0.62	0.360-1.059	0.082
Adjuvant chemotherapy (Yes vs No)	0.579	0.414-0.806	0.001	0.444	0.273-0.715	<0.001
R status(R1vsR0)	3.861	2.338-6.490	<0.001	4.861	2.360-10.321	<0.001

### 3.3 Diagnostic nomogram development and validation

**Figure 2:**
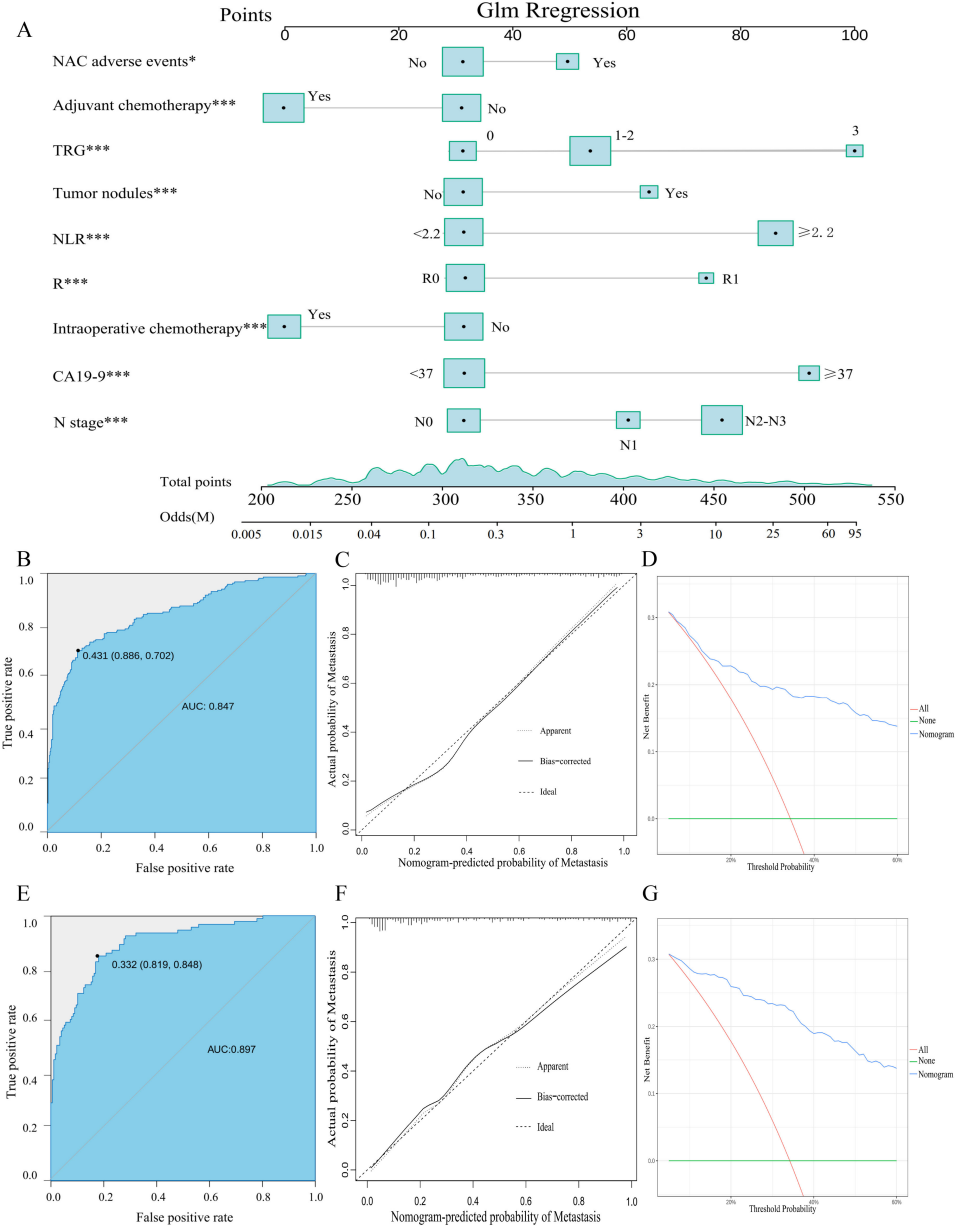
Development and validation of a risk nomogram for postoperative distant metastasis after neoadjuvant chemotherapy in elderly patients with locally advanced gastric adenocarcinoma. **(A)** Nomogram derived from multivariable logistic regression; **(B)** Receiver operating characteristic curve and area under the curve in the training cohort; **(C)** Calibration plot in the training cohort; **(D)** Decision curve analysis in the training cohort; **(E)** Receiver operating characteristic curve and area under the curve in the validation cohort; **(F)** Calibration plot in the validation cohort; **(G)** Decision curve analysis in the validation cohort.

**Figure 3:**
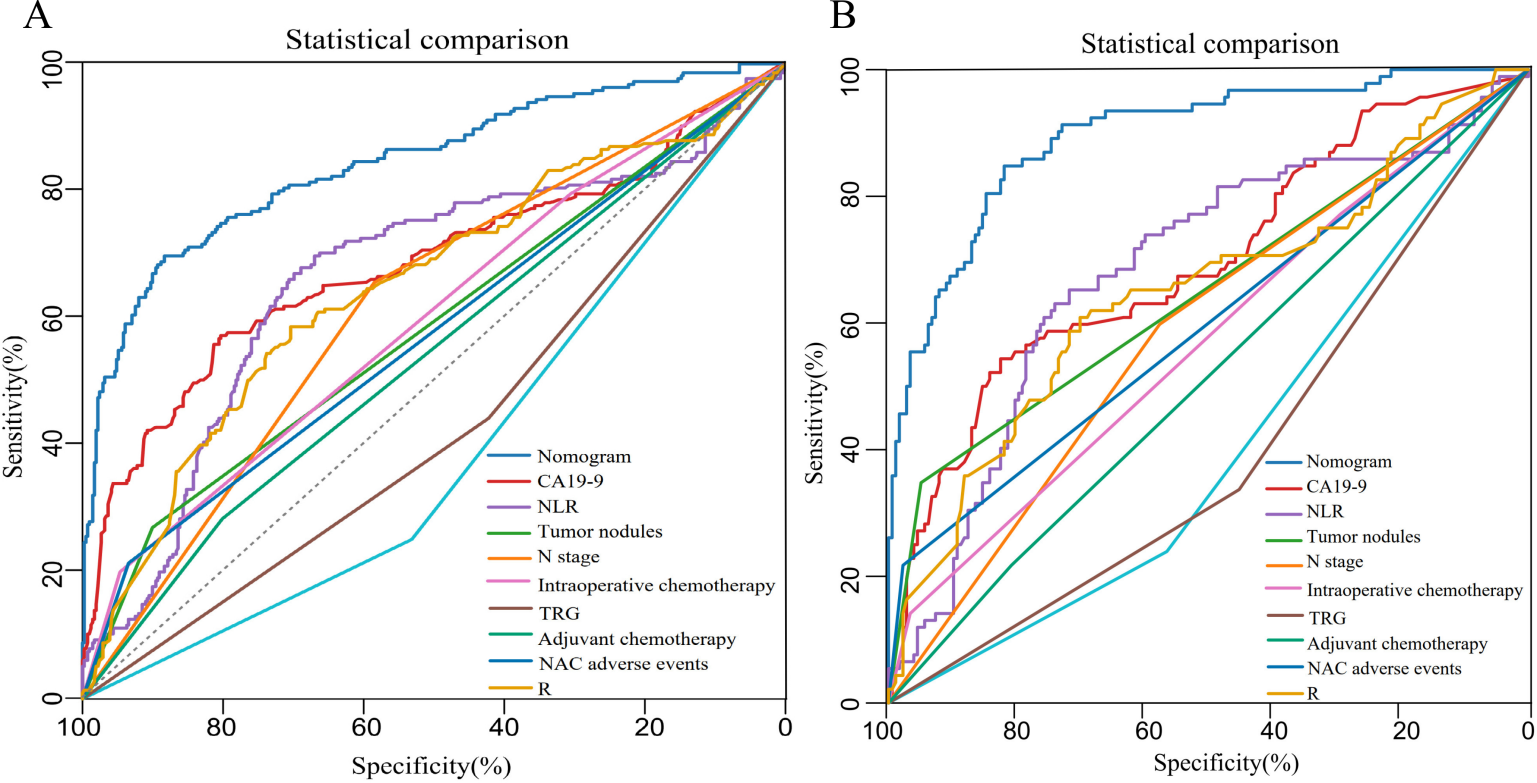
Comparison of ROC curves and AUCs between the nomogram and individual predictors in the training **(A)** and validation **(B)** cohorts. Individual predictors include CA19-9, NLR, tumor nodules, N stage, intraoperative chemotherapy, the tumor regression grade (TRG), adjuvant chemotherapy, NAC-related adverse events, and resection margin(R).

Based on the nine independent predictors identified, a diagnostic nomogram was constructed to predict the risk of postoperative distant metastasis in elderly patients with locally advanced gastric adenocarcinoma following neoadjuvant chemotherapy (**Figure 2A**). The incorporated variables included N stage, occurrence of NAC-related adverse events, CA19–9 level, neutrophil-to-lymphocyte ratio, presence of tumor nodules, tumor regression grade, resection margin status, intraoperative chemotherapy, and postoperative adjuvant chemotherapy. The nomogram demonstrated strong discriminatory power, with area under the curve values of 0.847 in the training set and 0.897 in the validation set, as evidenced by the ROC curves (**Figures 2B, E**). For comparative purposes, ROC curves for each individual predictor were also generated (**Figures 3A, B**). The predictive model exhibited superior performance in distinguishing patients with and without DM compared to any single predictor in both cohorts. Furthermore, the calibration curves showed excellent agreement between the nomogram-predicted probabilities and the actual observed outcomes in both the training and validation sets (**Figures 2C, F**). Additionally, DCA revealed that the nomogram provides positive net clinical benefit across a reasonable range of risk thresholds, suggesting its potential value for clinical application as a precise tool for DM risk assessment (**Figures 2D, G**).

### 3.4 Prognostic factors in patients with postoperative distant metastasis

Among the 307 enrolled elderly patients with locally advanced gastric adenocarcinoma who developed postoperative distant metastasis, a 7:3 random allocation was performed to create a training set and a validation set. No statistically significant differences in baseline characteristics were observed between the two sets (**Supplementary Table 3**). Linearity testing confirmed that all continuous variables satisfied the linearity assumption for inclusion in the Cox regression model (**Supplementary Table 4**). Univariate and multivariate Cox regression analyses were conducted in the prognostic cohort, with complete results shown in **Supplementary Table 5**. The multivariate model identified the following as independent predictors of poorer overall survival: elevated CA72–4 level (HR = 1.024, 95% CI: 1.010–1.030, P < 0.001), elevated neutrophil-to-lymphocyte ratio (NLR) (HR = 1.634, 95% CI: 1.170–1.690, P < 0.001), prolonged interval between the last NAC cycle and surgery (HR = 1.042, 95% CI: 1.010–1.060, P = 0.001), higher tumor regression grade (TRG 2/3 vs. TRG 0), and positive resection margins (P < 0.05). In contrast, postoperative chemotherapy was a protective factor (HR = 0.382, 95% CI: 0.257–0.568, P < 0.001). These results indicate that inflammation-related markers, pathological response to NAC, and resection margin status are significant predictors of survival in elderly patients with metastasis. Furthermore, standardized postoperative chemotherapy may help improve prognosis (**Table 3**).

**Table 3:** Univariate and multivariable Cox analyses of overall survival in elderly gastric cancer patients with postoperative distant metastasis.

Variable (comparison)	Univariate analysis			Multivariate analysis		
	HR	95% CI	P	HR	95% CI	P
CA19-9(≥37 vs<37)	1.573	1.136-2.179	0.006	1.423	0.987-2.030	0.059
CA72-4(≥7 vs<7)	1.556	1.124-2.156	0.008	1.572	1.083-2.286	<0.017
Neutrophils (per unit)	1.112	1.066-1.160	<0.001	0.938	0.859-1.020	0.154
NLR (per unit)	1.542	1.3340-1.781	<0.001	1.634	1.17-1.690	<0.001
SII (per unit)	1.002	1.001-1.003	<0.001	1.001	0.995-1.014	0.184
Interval to surgery (per 1 day)	1.033	1.010-1.056	0.004	1.042	1.010-1.060	0.001
TRG (1 vs 0)	1.520	0.975-2.369	0.064	1.471	0.927-2.362	0.107
TRG (2 vs 0)	1.683	1.024-2.431	0.033	1.563	1.293-2.640	0.043
TRG (3 vs 0)	1.748	1.058-2.889	0.029	1.793	1.074-3.013	0.027
Adjuvant chemotherapy (Yes vs No)	0.316	0.216-0.463	<0.001	0.382	0.257-0.568	<0.001
R status (R1 vs R0)	2.658	1.875-3.767	<0.001	2.396	1.647-3.493	<0.001

### 3.5 Prognostic nomogram development and validation

**Figure 4:**
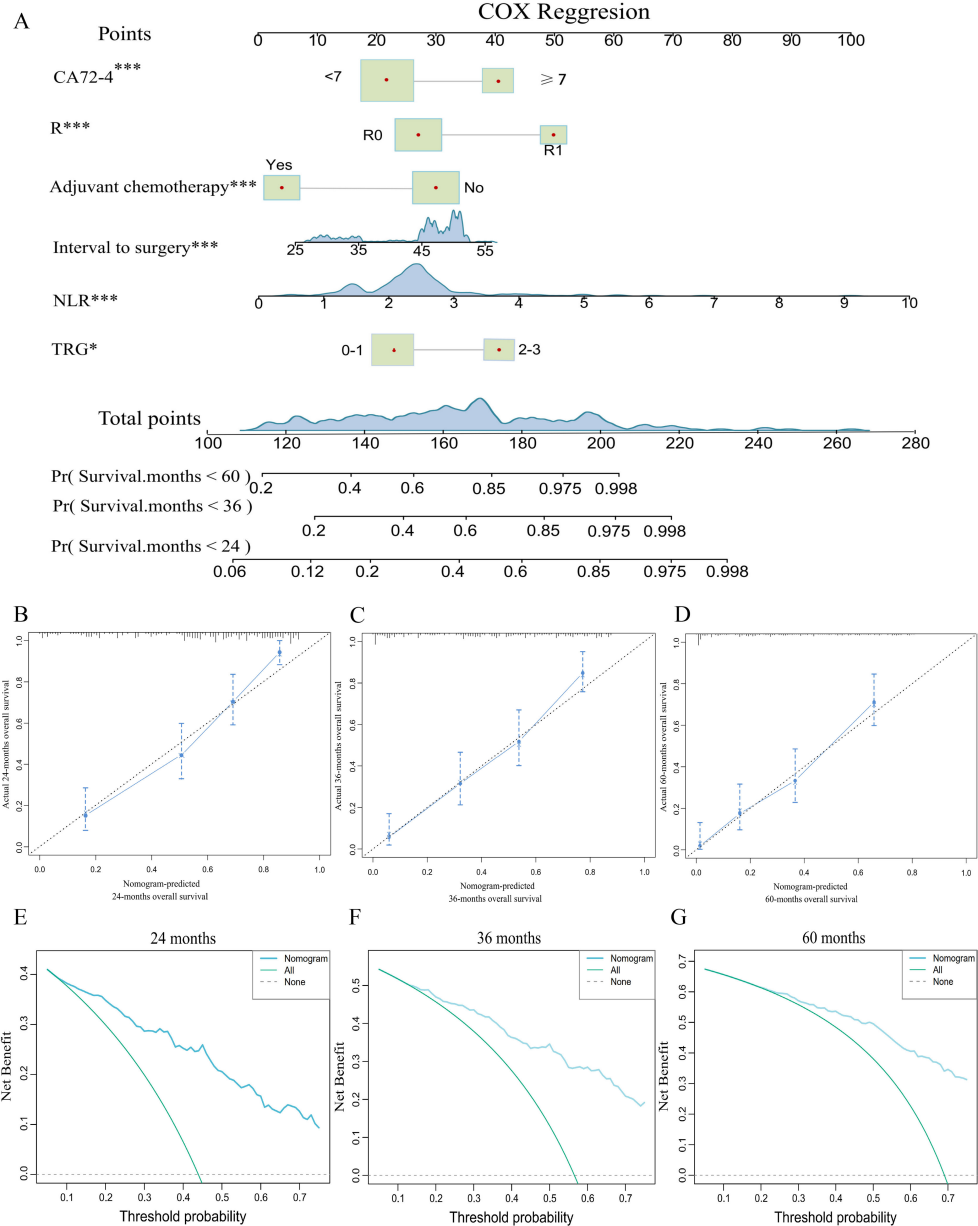
Prognostic nomogram for OS in elderly gastric cancer patients with postoperative metastasis. **(A)** Nomogram based on Cox analysis; **(B-D)** Calibration curves at 24, 36, and 60 months (training cohort); **(E-G)** Decision curve analysis at corresponding timepoints (training cohort).

**Figure 5:**
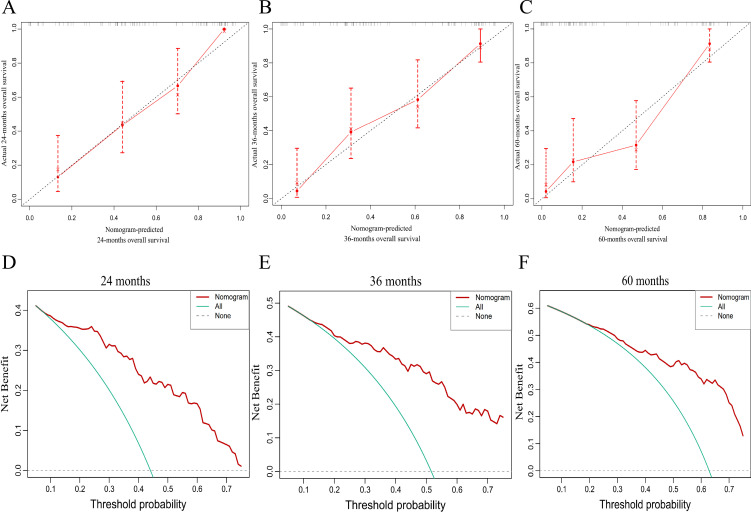
Calibration and decision-curve analyses of the nomogram in the validation cohort. **(A)** 24 months, **(B)** 36 months, and **(C)** 60 months calibration plots; **(D)** 24 months, **(E)** 36 months, and **(F)** 60 months decision-curve analyses.

**Figure 6:**
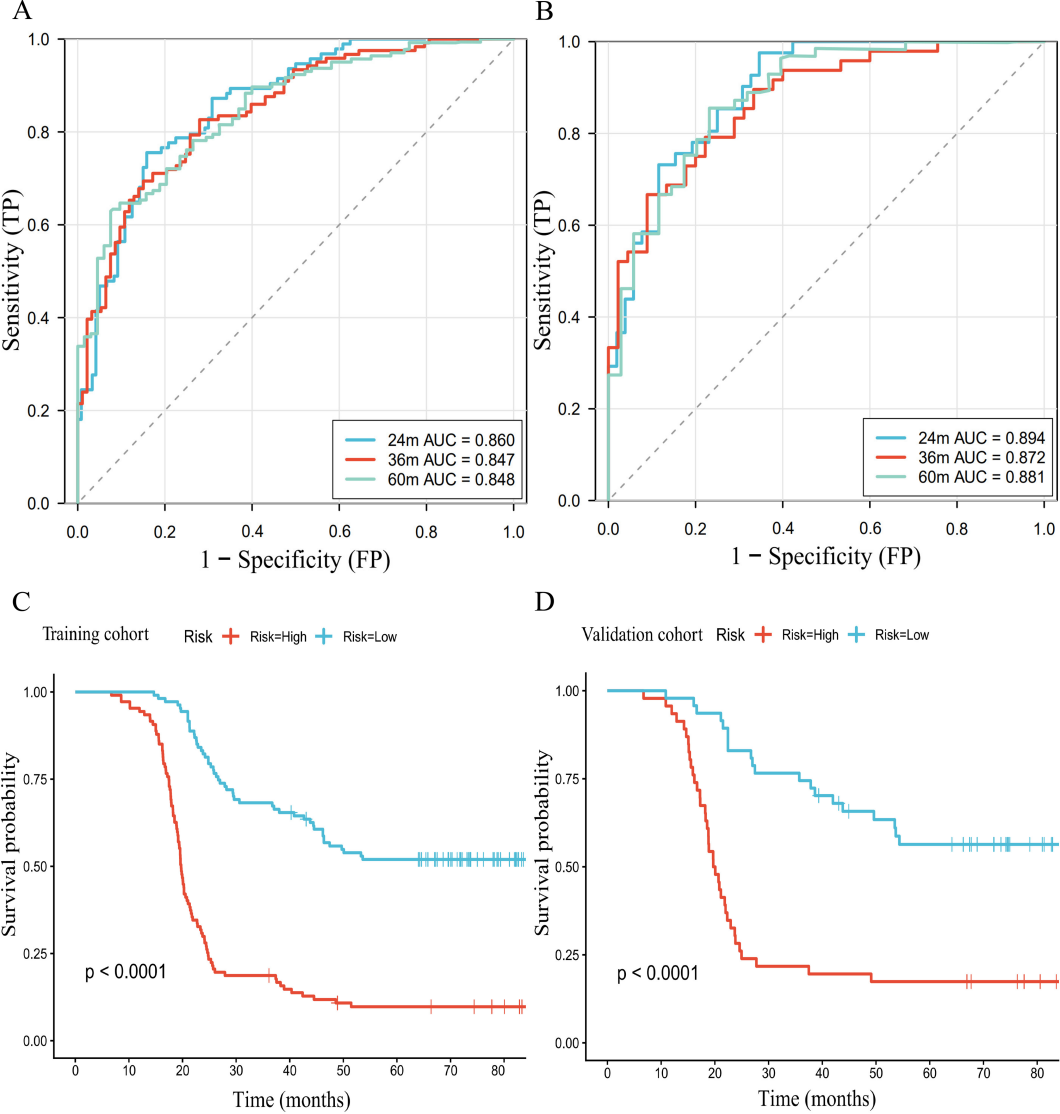
Time-dependent ROC curve analysis of the nomogram for the 24, 36, and 60 months in the training set **(A)** and the validation set **(B)**. The Kaplan Meier survival curves of the patients in the training set **(C)** and in the validation set **(D)**.

**Figure 7:**
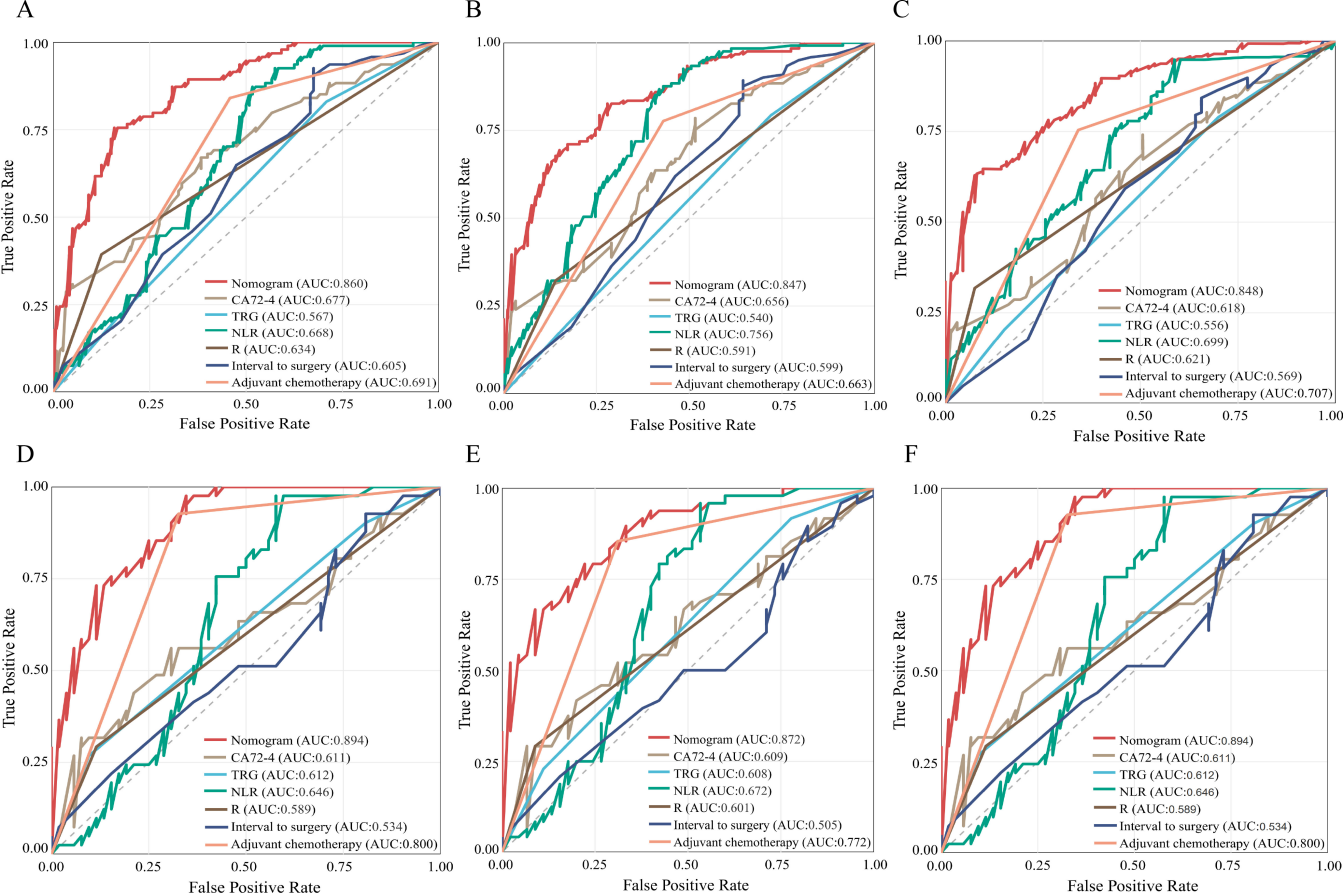
Comparison of ROC curves and AUCs between the nomogram and individual predictors (CA72-4, the tumor regression grade (TRG), NLR, resection margin (R), interval to surgery, and adjuvant chemotherapy) at 24 months **(A, D)**, 36 months **(B, E)**, and 60 months **(C, F)** in the training and validation cohorts.

Based on the six independent prognostic factors, a Cox nomogram was developed to predict overall survival in elderly patients with locally advanced gastric adenocarcinoma who developed postoperative distant metastasis (**Figure 4A**). The calibration curves for the predicted 24, 36, 60 months OS probabilities demonstrated strong agreement between the nomogram-predicted outcomes and the actual observations in both the training set (**Figures 4B-D**) and the validation set (**Figures 5A-C**). Furthermore, DCA confirmed the favorable clinical utility of the nomogram across a wide range of risk thresholds (**Figures 4E-G**, **5D-F**). ROC analysis indicated that the nomogram achieved AUC values of 0.860, 0.847, and 0.848 at 24, 36, and 60 months, respectively, in the training set (**Figure 6A**), and 0.894, 0.872, and 0.881 in the validation set (**Figure 6B**), reflecting its strong discriminative ability in predicting OS for this patient population. Kaplan-Meier survival analysis revealed that patients in the high-risk group had significantly poorer OS compared to those in the low-risk group (**Figures 6C, D**). Additionally, the nomogram demonstrated superior discriminative performance over any single independent prognostic factor at 24, 36, and 60 months, as evidenced by time-dependent ROC curve comparisons (**Figures 7A-F**).

## 4 Discussion

Gastric cancer imposes a substantial global disease burden due to its aggressive nature and poor prognosis, a concern that is particularly pronounced among the elderly population. However, high-quality evidence to guide treatment strategies in patients aged over 70 remains scarce. Importantly, therapeutic decisions in older adults should not rely solely on chronological age (15). Although existing studies indicate that the relative survival benefit from standard treatment is comparable between elderly and younger patients, older individuals face a higher risk of perioperative complications and therapy-related toxicities (16). Moreover, age-related physiological decline, comorbidities, and tumor-related factors—such as impaired gastric emptying, malabsorption, malnutrition, and sarcopenia—increase their susceptibility to dose reductions and cumulative toxicity during systemic anticancer treatment (17). For LAGC, NAC has become a cornerstone of perioperative management. Meta-analyses in Asian populations reveal that NAC significantly improves both disease-free survival and overall survival in elderly patients compared to surgery alone (18). Nonetheless, widespread adoption does not imply absence of risk. Marked interindividual variability exists among older patients in terms of tolerance and achievable dose intensity of NAC (2). Furthermore, even after completing NAC and achieving R0 resection, distant metastasis remains a leading cause of treatment failure, profoundly affecting long-term outcomes, with peritoneal and hepatic metastases being especially frequent (19). This underscores the urgent need to identify high-risk elderly subgroups predisposed to postoperative metastasis following NAC, which would allow tailored adjuvant strategies and intensified surveillance. Once DM develops, the absolute benefit that elderly patients gain from secondary surgeries, chemotherapy, or novel immunotherapies is often limited, and their prognosis tends to be worse than that of younger patients.

While several studies have explored prognostic predictors in gastric cancer, the majority have focused on the general population, with a paucity of dedicated analyses for elderly patients (20). Addressing this gap, our study developed and validated two specific tools for elderly patients with LAGC who received NAC followed by radical gastrectomy, a diagnostic nomogram for predicting the risk of postoperative DM and a prognostic nomogram for stratifying OS in those who developed DM. By incorporating readily accessible clinical variables, these nomograms generate individualized risk scores to inform decisions regarding postoperative surveillance intensity, consideration of intensified adjuvant therapy, and eligibility for clinical trials. Encouragingly, both models demonstrated robust and stable performance, with high AUC values, well-fitted calibration curves, and DCA indicating positive net clinical benefit across a wide range of risk thresholds. To our knowledge, this represents one of the largest studies specifically focused on predicting both “postoperative metastasis risk” and “post-metastasis survival” in this vulnerable population. Our models incorporate multidimensional variables-including laboratory indices, pathological grading, perioperative details, and chemotherapy-related data—collectively enhancing their general applicability and clinical utility.

NAC is widely considered a standard strategy for tumor downstaging and improving R0 resection rates in resectable LAGC. In our real-world elderly cohort, the specific NAC regimen or number of cycles did not show a significant independent impact on OS or DM. Previous research suggests that triplet regimens like docetaxel, oxaliplatin, and capecitabine DOX may offer superior 3-year OS and higher pathological complete response rates compared to doublets like XELOX, indicating potential biological advantage (21). However, in the clinical reality of elderly patients, this intensity benefit is often counterbalanced by tolerability issues and reduced treatment completion (22). Our finding that NAC-related adverse events independently correlated with increased DM risk underscores this trade-off. Treatment modifications (dose reduction, delay, or discontinuation) due to severe toxicity can compromise systemic control of micrometastases, increasing the risk of subsequent relapse. These findings underscore the importance of individualized dosing and proactive supportive care, including early nutritional and anti-sarcopenia interventions, primary prophylaxis with G-CSF for high-risk individuals, standardized antiemetic regimens, and comprehensive geriatric assessment (23). Ultimately, balancing regimen efficacy with personalized management is paramount, and overcoming chemoresistance and improving toxicity management remain critical challenges for future research.

The NLR, a readily available hematological marker reflecting systemic inflammation and immune suppression, emerged as a significant factor in both our DM risk and post-DM OS models, suggesting its role throughout the disease continuum from recurrence to progression and death. This aligns with numerous studies linking elevated NLR to worse OS and PFS (24). Neutrophils and tumor-associated neutrophils can promote angiogenesis, migration, and epithelial-mesenchymal transition through the secretion of factors like VEGF, MMP-9, and IL-17 (25), with the IL-17A–JAK2/STAT3 axis directly implicated in gastric cancer cells (26). Additionally, neutrophil extracellular traps can induce DNA damage/genomic instability and foster tumor progression and angiogenesis. Gastric cancer-derived extracellular vesicles can further activate and polarize neutrophils via the HMGB1/TLR4/NF-κB pathway, amplifying these effects (27). Regarding serum tumor markers, CA19–9 and CA72–4 showed distinct associations in our models, with CA19–9 linked to “DM risk” and CA72–4 to “OS after DM,” suggesting a meaningful division of labor. Previous evidence indicates that CA19–9 often rises with increasing tumor burden/dissemination risk and is associated with a higher likelihood of liver/peritoneal metastases (28). CA72-4, in contrast, is a mucin-related marker with greater specificity for gastric tissue. Its levels are particularly elevated in advanced or diffuse-type diseases that exhibit serosal or peritoneal spread, which likely accounts for its stronger prognostic signal in the overall survival model during the metastatic phase (29). The TRG assesses tumor response following NAC. When combined with the ypN stage, it significantly improves prognostic discrimination, prompting the proposal of integrated staging systems that incorporate both factors to refine survival prediction in gastric cancer (30). Our study corroborates this, demonstrating a stepwise decline in survival with higher TRG and ypN stage. This is consistent with the biological rationale that chemotherapy-resistant clones are more likely to survive treatment, seed micrometastases, and ultimately lead to clinical recurrence. While NAC is designed to eradicate micrometastases preoperatively and reduce recurrence risk (31), literature suggests that non-responders derive little improvement in distant metastasis-free survival even from adjuvant chemotherapy, implying that resistant clones may have already “escaped” during the neoadjuvant phase (32). Furthermore, an R1 resection margin can be viewed as direct evidence of “local microscopic residual disease leading to reseeding,” explaining its adverse impact in both our DM risk and OS models. Previous data also consistently show shorter OS for R1 patients compared to R0 (33).

The protective effects observed for both intraoperative intraperitoneal chemotherapy and postoperative adjuvant chemotherapy in our models align with the clinical reality that peritoneal recurrence is a common failure pattern in gastric cancer (34). It is important to note that conclusions from prior randomized trials have been inconsistent, and significant variations exist in techniques, drug selection, and patient criteria across centers, contributing to heterogeneous overall evidence. However, the “protective signal” observed in our real-world data is noteworthy. For patients at high risk of peritoneal recurrence (e.g., T3/T4, poorly differentiated) but without established DM, intraoperative intraperitoneal chemotherapy might be considered a preventive strategy (35). Evidence from colorectal cancer suggests that prophylactic intraoperative intraperitoneal chemotherapy can reduce the risk of subsequent peritoneal metastases, providing a rationale for cross-cancer strategy translation, though high-quality prospective data specific to gastric cancer are still needed (36). Postoperative adjuvant chemotherapy remains a critical component, with standard regimens and full course completion being essential. While delays in initiating adjuvant therapy have been associated with poorer outcomes, subsequent full-course completion can partially mitigate this negative impact (37). Given that elderly patients are more prone to incomplete treatment due to declining performance status, comorbidities, or frailty, a pragmatic approach involving dose individualization and intensive supportive care is crucial to balance relative dose intensity against severe toxicity, thereby maximizing net clinical benefit.

In the overall survival model for the distant metastasis cohort, a prolonged interval between neoadjuvant chemotherapy completion and surgery was independently associated with worse survival. This suggests that the time interval itself may represent a modifiable risk factor—an often-overlooked form of “time toxicity.” Particularly for patients with a poor response to NAC, delaying surgery risks missing the optimal window for resection while the patient continues to endure the side effects of chemotherapy and potential functional decline. Therefore, the decision to extend this interval must be guided by individualized response assessment and multidisciplinary discussion, carefully weighing the potential for further tumor regression against the risks of disease progression and dissemination (38). It is critical to emphasize the current absence of high-quality prospective trials directly comparing standardized intervals (e.g., 4 versus 8 weeks). Current evidence is largely derived from retrospective studies or combined-modality trials, wherein it is notoriously difficult to isolate the effect of the interval from the underlying tumor biology and treatment response. Consequently, in clinical practice, the strategy of intentionally extending the surgery interval should be approached with caution and pursued only alongside careful, dynamic monitoring of tumor response and resectability.

This study has several limitations. First, the relatively limited cohort of elderly patients with distant metastases from locally advanced gastric adenocarcinoma (N = 307) may have contributed to potential errors in the model. Second, although our nomograms were constructed using a training set and validated internally, external validation in independent cohorts is necessary to confirm their generalizability and mitigate inherent biases. Third, while we included key clinical, pathological, and laboratory variables, several important prognostic factors were not systematically integrated into the model, namely molecular markers, radiomic features, and comprehensive geriatric assessments. Finally, as a retrospective analysis, using “regimen/cycles” as exposure variables may not fully capture the impact of actual relative dose intensity, treatment delays, and dose modifications, potentially leading to an underestimation of how treatment intensity and completion influence outcomes. Future research should focus on prospective validation and incorporate these additional biomarkers, radiomic features, and comprehensive geriatric assessments, along with more granular treatment data, to refine predictive models. We believe that such advancements will significantly contribute to personalized perioperative management strategies and improve long-term outcomes in elderly patients with locally advanced gastric cancer (LAGC).

## 5 Conclusion

This study addresses the critical clinical challenge of predicting postoperative distant metastasis risk and individualized prognosis after metastasis in elderly patients with locally advanced gastric cancer following neoadjuvant chemotherapy, for whom specialized predictive tools are currently lacking. Through a retrospective analysis of 896 patients over 70 years of age, the study developed and validated two novel nomograms specifically for this population: the first model successfully identified eight independent risk factors for postoperative distant metastasis, including N stage, NAC-related adverse events, and CA19–9 levels; the second model, focusing on the 307 patients who developed metastasis, identified six independent prognostic factors, such as CA72–4 levels, the interval from NAC to surgery, and tumor regression grade. Validation results demonstrated that both nomograms exhibit exceptional predictive accuracy and clinical applicability. These tools enable the early identification of high-risk patients for metastasis from the preoperative to postoperative phases, guiding intensified monitoring and adjuvant therapy, while also providing individualized prognosis assessment after metastasis confirmation. This research offers a practical and innovative solution for advancing precision clinical decision-making in the management of elderly gastric cancer patients.

## Data Availability

The original contributions presented in the study are included in the article/**Supplementary Material**. Further inquiries can be directed to the corresponding authors.

## References

[1] Sundar R Nakayama I Markar SR Shitara K van Laarhoven HWM Janjigian YY . Gastric cancer. Lancet. (2025) 405:2087–102. doi: 10.1016/S0140-6736(25)00052-2, PMID: 40319897

[2] Keywani K Borgstein ABJ Eshuis WJ Pape M Versteeg KS Derks S . Neoadjuvant chemotherapy in older patients with gastric cancer undergoing surgery: a population-based cohort study. Gastric Cancer. (2023) 26:763–74. doi: 10.1007/s10120-023-01404-2, PMID: 37285071 PMC10361849

[3] de la Fouchardiere C Decoster L Samalin E Terret C Kenis C Droz JP . Advanced esophago-gastric adenocarcinoma in older patients in the era of immunotherapy. A review of the literature. Cancer Treat Rev. (2021) 100:102289. doi: 10.1016/j.ctrv.2021.102289, PMID: 34583303

[4] Zhu W Dong W Liu Y Bai R . Stomach cancer epidemic in Chinese mainland: Current trends and future predictions. Chin Med J (Engl). (2025) 138:205–12. doi: 10.1097/CM9.0000000000002993, PMID: 39157911 PMC11745847

[5] Hu J Li X Wang Y Xu B He P Wang Z . SOX combined with apatinib and camrelizumab in the treatment of resectable locally advanced gastric cancer: a case report. Front Immunol. (2024) 15:1410284. doi: 10.3389/fimmu.2024.1410284, PMID: 39072331 PMC11272450

[6] Rawicz-Pruszyński K Endo Y Tsilimigras DI Munir MM Resende V Kim A . Neoadjuvant chemotherapy improves oncological outcomes and long-term survival among elderly patients with locally advanced gastric cancer: A propensity score matched analysis. Ann Surg Oncol. (2024) 31:753–61. doi: 10.1245/s10434-023-14569-y, PMID: 37985525

[7] Shih YH Lin HC Liao PW Chou CW Lin CH Hsu CY . The efficacy of adjuvant chemotherapy for older adults with stage II/III gastric cancer: a retrospective cohort study. BMC Cancer. (2023) 23:770. doi: 10.1186/s12885-023-11244-z, PMID: 37596599 PMC10436551

[8] Szabo Yamashita T Williams-Perez SM Ehsan S . The multi-institutional medullary thyroid cancer collaborative registry: can a rare tumor registry accurately represent the real-world patient population? Thyroid. (2024) 34:1117–25. doi: 10.1089/thy.2024.0239, PMID: 38984944 PMC11698660

[9] Shiels MS Freedman ND Haque AT Berrington de González A Lipkowitz S Lowy DR . cancer deaths prevented due to survival improvements stratified by extent of disease, 2010-2019. J Natl Cancer Inst. (2025) 16:djaf192. doi: 10.1093/jnci/djaf192, PMID: 40668759 PMC12505133

[10] Huang X Li Z Weng Q . Clinicopathological features and prognostic significance of site-specific metastasis in gastric cancer: a population-based, propensity score-matched analysis. Discov Oncol. (2025) 16:1164. doi: 10.1007/s12672-025-02865-w, PMID: 40540159 PMC12181138

[11] Luan X Han X Wang Z Zhao L Zhang X Wang W . Heterogeneity of metastatic gastric cancer: solitary non-regional lymph node metastasis and solitary lung metastasis showed better survival outcomes than other metastatic patterns. BMC Cancer. (2025) 25:1287. doi: 10.1186/s12885-025-14748-y, PMID: 40781666 PMC12333190

[12] Robella M Vitturini M Di Giorgio A Aulicino M Hubner M Koumantakis E . Advances in bidirectional therapy for peritoneal metastases: A systematic review of pressurized intraperitoneal aerosol chemotherapy (PIPAC) combined with systemic chemotherapy. Cancers (Basel). (2025) 17:2580. doi: 10.3390/cancers17152580, PMID: 40805276 PMC12345692

[13] Gao P Xiao Q Tan H Song J Fu Y Xu J . Interpretable multi-modal artificial intelligence model for predicting gastric cancer response to neoadjuvant chemotherapy. Cell Rep Med. (2024) 5:101848. doi: 10.1016/j.xcrm.2024.101848, PMID: 39637859 PMC11722130

[14] Lee HI . Relationships between microbiome and response to neoadjuvant chemoradiotherapy in rectal cancer. Int J Radiat oncol biol Phys. (2023) 57(3):840–51. doi: 10.1016/S0167-8140(23)08571-7, PMID: 39701092 PMC12263241

[15] Rawla P Barsouk A . Epidemiology of gastric cancer: global trends, risk factors and prevention. Prz Gastroenterol. (2019) 14:26–38. doi: 10.5114/pg.2018.80001, PMID: 30944675 PMC6444111

[16] Merga ZC Lee JS Gong CS . Outcomes of gastrectomy for gastric cancer in patients aged >80 years: A systematic literature review and meta-analysis. J Gastric Cancer. (2023) 23:428–50. doi: 10.5230/jgc.2023.23.e23, PMID: 37553130 PMC10412976

[17] Hamaker ME Oosterlaan F van Huis LH Thielenc N Vondelinga A van den Bosb F . Nutritional status and interventions for patients with cancer - A systematic review. J Geriatr Oncol. (2021) 12:6–21. doi: 10.1016/j.jgo.2020.06.020, PMID: 32616384

[18] Chang SH Kim SN Choi HJ Park M Kim RB Go SI . Adjuvant chemotherapy for advanced gastric cancer in elderly and non-elderly patients: meta-analysis of randomized controlled trials. Cancer Res Treat. (2017) 49:263–73. doi: 10.4143/crt.2016.054, PMID: 27384158 PMC5266393

[19] Hayashi M Fujita T Matsushita H . Prognostic relevance of recurrent sites of gastric cancer treated with curative resection: A single center retrospective study. J Gastric Cancer. (2024) 24:291–9. doi: 10.5230/jgc.2024.24.e23, PMID: 38960888 PMC11224719

[20] Zhao L Huang H Zhao D Wang C Tian Y Yuan X . Clinicopathological characteristics and prognosis of proximal and distal gastric cancer during 1997–2017 in China national cancer center. J Oncol. (2019) 2019:9784039. doi: 10.1155/2019/9784039, PMID: 31312217 PMC6595386

[21] Gu T Wang Y Wu Z He N Li Y Shan F . Feasibility and long-term survival of proximal gastrectomy after neoadjuvant therapy for locally advanced proximal gastric cancer: A propensity-score-matched analysis. Chin Med J (Engl). (2025) 138:1984–90. doi: 10.1097/CM9.0000000000003232, PMID: 39090777 PMC12369804

[22] Wang J Wu Z de Groot EM Challine A Mohammad NH Mook S . Discontinuation of neoadjuvant therapy does not influence postoperative short-term outcomes in elderly patients (≥ 70 years) with resectable gastric cancer: a population-based study from the dutch upper gastrointestinal cancer audit (DUCA) data. Gastric Cancer. (2024) 27:1114–23. doi: 10.1007/s10120-024-01522-5, PMID: 38918269 PMC11335952

[23] Kobayshi K Suyama K Katsuya H Izawa N Uenosono Y Hu Q . A phase II multicenter trial assessing the efficacy and safety of first-line S-1 + ramucirumab in elderly patients with advanced/recurrent gastric cancer: KSCC1701. Eur J Cancer. (2022) 166:279–86. doi: 10.1016/j.ejca.2022.02.028, PMID: 35349925

[24] Tan S Zheng Q Zhang W Zhou M Xia C Feng W . Prognostic value of inflammatory markers NLR, PLR, and LMR in gastric cancer patients treated with immune checkpoint inhibitors: a meta-analysis and systematic review. Front Immunol. (2024) 15:1408700. doi: 10.3389/fimmu.2024.1408700, PMID: 39050856 PMC11266030

[25] Wu Y Zhao Q Peng C Sun L Li XF Kuang DM . Neutrophils promote motility of cancer cells via a hyaluronan-mediated TLR4/PI3K activation loop. J Pathol. (2011) 225:438–47. doi: 10.1002/path.2947, PMID: 21826665

[26] Butin-Israeli V Bui TM Wiesolek HL Mascarenhas L Lee JJ Mehl LC . Neutrophil-induced genomic instability impedes resolution of inflammation and wound healing. J Clin Invest. (2019) 129:712–26. doi: 10.1172/JCI122085, PMID: 30640176 PMC6355304

[27] Zhang Y Guo L Li Y Feng GH Teng F Li W . MicroRNA-494 promotes cancer progression and targets adenomatous polyposis coli in colorectal cancer. Mol Cancer. (2018) 17:1. doi: 10.1186/s12943-017-0753-1, PMID: 29304823 PMC5755155

[28] Lee T Teng TZJ Shelat VG . Carbohydrate antigen 19-9 - tumor marker: Past, present, and future. World J Gastrointest Surg. (2020) 12:468–90. doi: 10.4240/wjgs.v12.i12.468, PMID: 33437400 PMC7769746

[29] Xu Y Zhang P Zhang K Huang C . The application of CA72–4 in the diagnosis, prognosis, and treatment of gastric cancer. Biochim Biophys Acta Rev Cancer. (2021) 1876:188634. doi: 10.1016/j.bbcan.2021.188634, PMID: 34656687

[30] Weng CM Zhong Q Sun YQ Liu ZY Ma YB Zhang ZQ . A novel ypN-TRG staging system for gastric cancer patients after neoadjuvant therapy based on the metro-ticket paradigm: a multicenter and large sample retrospective analysis. Gastric Cancer. (2025) 28:465–77. doi: 10.1007/s10120-025-01586-x, PMID: 39918688

[31] Turri G Ostuzzi G Vita G Barresi V Scarpa A Milella M . Treatment of locally advanced rectal cancer in the era of total neoadjuvant therapy: A systematic review and network meta-analysis. JAMA Netw Open. (2024) 7:e2414702. doi: 10.1001/jamanetworkopen.2024.14702, PMID: 38833249 PMC11151159

[32] Yang Y He Y Fan Z Chen X Liu Y Zhang C . Phase III study of HR-positive/HER2-negative/lymph node-positive breast cancer non-responsive to primary chemotherapy: a randomized trial. NPJ Breast Cancer. (2023) 9:54. doi: 10.1038/s41523-023-00553-y, PMID: 37344451 PMC10284834

[33] Koh CE Brown KGM Steffens D . What constitutes a clear margin in patients with locally recurrent rectal cancer undergoing pelvic exenteration? Ann Surg. (2022) 275:157–65. doi: 10.1097/SLA.0000000000003834, PMID: 32068551

[34] Li GZ Doherty GM Wang J . Surgical management of gastric cancer: A review. JAMA Surg. (2022) 157:446–54. doi: 10.1001/jamasurg.2022.0182, PMID: 35319717

[35] Guchelaar NAD Nasserinejad K Mostert B Koolen SLW van der Sluis PC Lagarde SM . Intraperitoneal chemotherapy for peritoneal metastases of gastric origin: a systematic review and meta-analysis. Br J Surg. (2024) 111:znae116. doi: 10.1093/bjs/znae116, PMID: 38722803 PMC11081074

[36] Su H Zhang R Li Y Li Y Pei W Jie Z . Intraoperatively preventive intraperitoneal perfusion chemotherapy with lobaplatin in colorectal cancer: a prospective, randomized, controlled, multicenter study. BMC Med. (2025) 23:336. doi: 10.1186/s12916-025-04180-1, PMID: 40481437 PMC12142866

[37] Zhong Q Liu ZY Shang-Guan ZX Li YF Li Y Wu J . Impact of chemotherapy delay on long-term prognosis of laparoscopic radical surgery for locally advanced gastric cancer: a pooled analysis of four randomized controlled trials. Gastric Cancer. (2024) 27:1100–13. doi: 10.1007/s10120-024-01513-6, PMID: 38809487

[38] Zhou Z Ren Y Zhang Z Guan T Wang Z Chen W . Digital histopathological images of biopsy predict response to neoadjuvant chemotherapy for locally advanced gastric cancer. Gastric Cancer. (2023) 26:734–42. doi: 10.1007/s10120-023-01407-z, PMID: 37322381

