# Supplementary material for: Distant metastasis risk and prognosis in elderly gastric cancer patients after neoadjuvant chemotherapy and surgery
Source: Front Oncol. 2026 Feb 6;16:1757874. doi: 10.3389/fonc.2026.1757874 (PMC12920236; doi:10.3389/fonc.2026.1757874)
Supplement: Supplementary file 2 [file Table1.docx]

| Variable | Overall χ² | df | P | Nonlinear χ² | df | P | Determination |
| --- | --- | --- | --- | --- | --- | --- | --- |
| BMI | 4.24 | 3 | 0.2371 | 4.17 | 2 | 0.1244 | Approx. linear |
| Age | 1.99 | 3 | 0.575 | 1.93 | 2 | 0.3813 | Approx. linear |
| CEA | 7.38 | 3 | 0.0608 | 6.54 | 2 | 0.0381 | Nonlinear |
| CA19-9 | 87.99 | 3 | <0.0001 | 9.58 | 2 | 0.0083 | Nonlinear |
| CA72-4 | 46.03 | 3 | <0.0001 | 14.5 | 2 | 0.0007 | Nonlinear |
| CA125 | 46.89 | 3 | <0.0001 | 4.36 | 2 | 0.1131 | Approx. linear |
| Hemoglobin | 0.42 | 3 | 0.9352 | 0.13 | 2 | 0.9374 | Approx. linear |
| WBC | 6.81 | 3 | 0.0781 | 5.23 | 2 | 0.0732 | Borderline |
| Neutrophils | 25.31 | 3 | <0.0001 | 20.68 | 2 | <0.0001 | Nonlinear |
| Platelets | 9.34 | 3 | 0.0251 | 5.66 | 2 | 0.0589 | Borderline |
| Albumin | 14.65 | 3 | 0.0021 | 5.25 | 2 | 0.0725 | Borderline |
| Lymphocytes | 6.81 | 3 | 0.0781 | 5.23 | 2 | 0.0732 | Borderline |
| PLR | 4.94 | 3 | 0.1763 | 4.05 | 2 | 0.132 | Approx. linear |
| NLR | 66.25 | 3 | <0.0001 | 33.81 | 2 | <0.0001 | Nonlinear |
| SII | 29.16 | 3 | <0.0001 | 5.7 | 2 | 0.0579 | Borderline |
| Interval Surgery Time | 3.86 | 3 | 0.2765 | 0.35 | 2 | 0.8396 | Approx. linear |
| Supplementary Table S1 | | | | | | | |

| Variable (comparison) | Term | OR | 95% CI | P-value |
| --- | --- | --- | --- | --- |
| Sex | Female vs Male | 1.045 | 0.712–1.522 | 0.821 |
| Age (per year) | per 1 year | 0.994 | 0.957–1.033 | 0.778 |
| T stage (ref=T1) | T2 vs T1 | 0.348 | 0.013–9.024 | 0.461 |
| T stage (ref=T1) | T3 vs T1 | 0.492 | 0.019–12.540 | 0.618 |
| T stage (ref=T1) | T4 vs T1 | 0.823 | 0.032–21.077 | 0.891 |
| N stage (ref=N0) | N1 vs N0 | 1.753 | 1.022–2.994 | 0.04 |
| N stage (ref=N0) | N2 vs N0 | 3.895 | 2.458–6.246 | <0.001 |
| N stage (ref=N0) | N3 vs N0 | 2.821 | 1.787–4.497 | <0.001 |
| Tumor location (ref=Antrum) | Cardia vs Antrum | 0.71 | 0.479–1.051 | 0.087 |
| Tumor location (ref=Antrum) | Corpus vs Antrum | 0.809 | 0.521–1.250 | 0.342 |
| Tumor location (ref=Antrum) | Fundus vs Antrum | 0.668 | 0.206–1.871 | 0.463 |
| Tumor location (ref=Antrum) | Pylorus vs Antrum | 1.122 | 0.394–3.035 | 0.823 |
| NAC regimen (ref=FLOT) | FOLFOX vs FLOT | 1.327 | 0.629–2.806 | 0.457 |
| NAC regimen (ref=FLOT) | Oral vs FLOT | 1.04 | 0.545–1.988 | 0.904 |
| NAC regimen (ref=FLOT) | SOX vs FLOT | 0.458 | 0.268–0.786 | 0.004 |
| NAC regimen (ref=FLOT) | XELOX vs FLOT | 0.811 | 0.449–1.467 | 0.486 |
| NAC regimen (ref=FLOT) | Other vs FLOT | 1.3 | 0.557–3.033 | 0.541 |
| NAC cycles (per cycle) | per cycle | 0.995 | 0.800–1.240 | 0.966 |
| Diabetes (ref=No) | Yes vs No | 0.981 | 0.632–1.501 | 0.929 |
| Stroke history (ref=No) | Yes vs No | 1.411 | 0.914–2.163 | 0.116 |
| Coronary artery disease (ref=No) | Yes vs No | 0.905 | 0.514–1.548 | 0.722 |
| Hypertension (ref=No) | Yes vs No | 0.786 | 0.562–1.096 | 0.157 |
| BMI (per kg/m²) | per 1 kg/m² | 0.993 | 0.946–1.042 | 0.779 |
| Marital status (ref=Single/Divorced) | Married vs Single/Divorced | 0.5 | 0.251–1.004 | 0.048 |
| Marital status (ref=Single/Divorced) | Unknown vs Single/Divorced | 0.588 | 0.157–2.002 | 0.407 |
| NAC adverse events (any) (ref=No) | Yes vs No | 1.594 | 1.085–2.335 | 0.017 |
| Intraoperative chemotherapy (ref=No) | Yes vs No | 0.381 | 0.263–0.545 | <0.001 |
| Hemoglobin (per g/L) | per 1 g/L | 0.998 | 0.991–1.005 | 0.584 |
| WBC (per ×10^9/L) | per 1 | 0.965 | 0.909–1.019 | 0.217 |
| Neutrophils (per ×10^9/L) | per 1 | 1.053 | 0.999–1.111 | 0.055 |
| Platelets (per ×10^9/L) | per 1 | 1.002 | 1.000–1.005 | 0.053 |
| Albumin (per g/L) | per 1 g/L | 0.944 | 0.910–0.979 | 0.002 |
| Lymphocytes (per ×10^9/L) | per 1 | 0.837 | 0.622–1.099 | 0.217 |
| PLR (per unit) | per unit | 1.002 | 0.998–1.007 | 0.311 |
| SII (per unit) | per unit | 1.002 | 1.001–1.003 | <0.001 |
| Interval to surgery (days) | per day | 1.02 | 0.999–1.042 | 0.059 |
| Surgical approach (ref=Laparoscopic) | Open vs Laparoscopic | 0.905 | 0.587–1.410 | 0.653 |
| Gastrectomy extent (ref=Proximal) | Total vs Proximal | 1.301 | 0.834–2.049 | 0.251 |
| Gastrectomy extent (ref=Proximal) | Distal vs Proximal | 1.658 | 1.068–2.603 | 0.026 |
| Blood loss (per mL) | per 1 mL | 1.001 | 1.000–1.002 | 0.218 |
| Operative time (per min) | per 1 min | 0.997 | 0.993–1.001 | 0.129 |
| Postoperative complications (ref=No) | Yes vs No | 1.419 | 0.998–2.013 | 0.051 |
| Vascular tumor thrombus (ref=No) | Yes vs No | 1.743 | 1.251–2.436 | 0.001 |
| Perineural invasion (ref=No) | Yes vs No | 1.983 | 1.421–2.775 | <0.001 |
| Tumor nodules (ref=No) | Yes vs No | 3.343 | 2.157–5.224 | <0.001 |
| Lauren type (ref=Unknown) | Mixed vs Unknown | 0.578 | 0.369–0.901 | 0.016 |
| Lauren type (ref=Unknown) | Diffuse vs Unknown | 1.196 | 0.766–1.869 | 0.431 |
| Lauren type (ref=Unknown) | Intestinal vs Unknown | 0.804 | 0.486–1.319 | 0.39 |
| Signet-ring component (ref=No) | Yes vs No | 2.132 | 1.403–3.240 | <0.001 |
| TRG (ref=C0) | C1 vs C0 | 1.45 | 0.942–2.251 | 0.094 |
| TRG (ref=C0) | C2 vs C0 | 1.294 | 0.796–2.109 | 0.299 |
| TRG (ref=C0) | C3 vs C0 | 5.597 | 3.051–10.536 | <0.001 |
| Histologic grade (ref=Undiff/Poor) | Well/Moderate vs Undiff/Poor | 0.516 | 0.360–0.732 | <0.001 |
| Adjuvant chemotherapy (ref=No) | Yes vs No | 0.579 | 0.414–0.806 | 0.001 |
| R status (ref=R0) | R1 vs R0 | 3.861 | 2.338–6.490 | <0.001 |
| CA19-9 | （≥37 vs<37） | 7.28 | 4.770-11.360 | <0.001 |
| CA125 | （≥35 vs<35） | 3.138 | 2.19-4.512 | <0.001 |
| CA72-4 | （≥7 vs<7） | 2.175 | 1.530-3.096 | <0.001 |
| NLR | （≥2.2 vs<2.2） | 4.667 | 3.290-6.671 | <0.001 |
| CEA | （≥5 vs<5） | 1.743 | 1.220-2.487 | 0.002 |
| Supplementary Table S2 | | | | |

| Variable | Level | Training (n, %) | Validation (n, %) | Overall (n, %) | Statistic | P-value |
| --- | --- | --- | --- | --- | --- | --- |
| Sex | Male | 151 (70.6%) | 69 (74.2%) | 220 (71.7%) | χ²=0.261 | 0.6092 |
|  | Female | 63 (29.4%) | 24 (25.8%) | 87 (28.3%) |  |  |
| Age (years) | Mean (SD) | 75.5 (4.44) | 75.4 (4.20) | 75.5 (4.36) | χ²=12.829 | 0.8472 |
|  | Median [Min, Max] | 75.0 [70.0, 94.0] | 75.0 [70.0, 93.0] | 75.0 [70.0, 94.0] | |  |
| T stage | T0 | 1 (0.5%) | 0 (0%) | 1 (0.3%) | χ²=7.587 | 0.1079 |
|  | T1 | 30 (14.0%) | 16 (17.2%) | 46 (15.0%) |  |  |
|  | T2 | 19 (8.9%) | 17 (18.3%) | 36 (11.7%) |  |  |
|  | T3 | 102 (47.7%) | 34 (36.6%) | 136 (44.3%) | |  |
|  | T4 | 62 (29.0%) | 26 (28.0%) | 88 (28.7%) |  |  |
| N stage | N0 | 39 (18.2%) | 28 (30.1%) | 67 (21.8%) | χ²=6.559 | 0.0874 |
|  | N1 | 35 (16.4%) | 9 (9.7%) | 44 (14.3%) |  |  |
|  | N2 | 77 (36.0%) | 32 (34.4%) | 109 (35.5%) | |  |
|  | N3 | 63 (29.4%) | 24 (25.8%) | 87 (28.3%) |  |  |
| Tumor location | Antrum | 87 (40.7%) | 31 (33.3%) | 118 (38.4%) | χ²=4.143 | 0.3871 |
|  | Cardia | 72 (33.6%) | 36 (38.7%) | 108 (35.2%) | |  |
|  | Corpus | 40 (18.7%) | 23 (24.7%) | 63 (20.5%) |  |  |
|  | Fundus | 6 (2.8%) | 1 (1.1%) | 7 (2.3%) |  |  |
|  | Pylorus | 9 (4.2%) | 2 (2.2%) | 11 (3.6%) |  |  |
| NAC regimen | FLOT | 30 (14.0%) | 11 (11.8%) | 41 (13.4%) | χ²=2.875 | 0.7192 |
|  | FOLFOX | 24 (11.2%) | 9 (9.7%) | 33 (10.7%) |  |  |
|  | Oral | 34 (15.9%) | 14 (15.1%) | 48 (15.6%) |  |  |
|  | SOX | 70 (32.7%) | 26 (28.0%) | 96 (31.3%) |  |  |
|  | XELOX | 42 (19.6%) | 24 (25.8%) | 66 (21.5%) |  |  |
|  | Other | 14 (6.5%) | 9 (9.7%) | 23 (7.5%) |  |  |
| NAC cycles | Mean (SD) | 3.14 (0.783) | 3.15 (0.820) | 3.15 (0.793) | χ²=2.372 | 0.4989 |
|  | Median [Min, Max] | 3.00 [1.00, 4.00] | 3.00 [1.00, 4.00] | 3.00 [1.00, 4.00] | |  |
| Diabetes | No | 175 (81.8%) | 74 (79.6%) | 249 (81.1%) | χ²=0.087 | 0.7679 |
|  | Yes | 39 (18.2%) | 19 (20.4%) | 58 (18.9%) |  |  |
| Stroke history | No | 176 (82.2%) | 74 (79.6%) | 250 (81.4%) | χ²=0.155 | 0.6937 |
|  | Yes | 38 (17.8%) | 19 (20.4%) | 57 (18.6%) |  |  |
| Coronary artery disease | No | 192 (89.7%) | 83 (89.2%) | 275 (89.6%) | χ²≈0 | 1 |
|  | Yes | 22 (10.3%) | 10 (10.8%) | 32 (10.4%) |  |  |
| Hypertension | No | 127 (59.3%) | 58 (62.4%) | 185 (60.3%) | χ²=0.137 | 0.7114 |
|  | Yes | 87 (40.7%) | 35 (37.6%) | 122 (39.7%) | |  |
| BMI (kg/m²) | Mean (SD) | 23.2 (3.45) | 22.8 (3.13) | 23.1 (3.35) | χ²=229.26 | 0.4642 |
|  | Median [Min, Max] | 22.6 [15.1, 35.2] | 22.6 [16.6, 29.3] | 22.6 [15.1, 35.2] | |  |
| Marital status | Divorced | 22 (10.3%) | 4 (4.3%) | 26 (8.5%) | χ²=3.132 | 0.3718 |
|  | Married | 99 (46.3%) | 45 (48.4%) | 144 (46.9%) | |  |
|  | Single | 88 (41.1%) | 41 (44.1%) | 129 (42.0%) | |  |
|  | Unknown | 5 (2.3%) | 3 (3.2%) | 8 (2.6%) |  |  |
| NAC adverse events (any) | No | 156 (72.9%) | 70 (75.3%) | 226 (73.6%) | χ²=0.085 | 0.77 |
|  | Yes | 58 (27.1%) | 23 (24.7%) | 81 (26.4%) |  |  |
| CEA | Mean (SD) | 8.24 (12.6) | 10.8 (17.9) | 9.01 (14.4) | χ²=120.47 | 0.5979 |
|  | Median [Min, Max] | 4.33 [0, 92.4] | 4.50 [1.00, 99.6] | 4.50 [0, 99.6] | |  |
| CA19-9 | Mean (SD) | 31.5 (27.6) | 29.2 (26.9) | 30.8 (27.3) | χ²=156.92 | 0.2726 |
|  | Median [Min, Max] | 20.3 [1.38, 93.8] | 20.0 [0, 90.0] | 20.3 [0, 93.8] | |  |
| CA125 | Mean (SD) | 30.1 (24.6) | 31.7 (25.0) | 30.6 (24.7) | χ²=89.683 | 0.9551 |
|  | Median [Min, Max] | 25.7 [0.150, 98.9] | 26.1 [0.280, 98.0] | 25.7 [0.150, 98.9] | |  |
| CA72-4 | Mean (SD) | 11.4 (16.1) | 10.9 (13.8) | 11.3 (15.4) | χ²=88.191 | 0.8332 |
|  | Median [Min, Max] | 4.41 [0.330, 69.8] | 4.08 [0.330, 58.5] | 4.41 [0.330, 69.8] | |  |
| Intraoperative chemotherapy | No | 163 (76.2%) | 68 (73.1%) | 231 (75.2%) | χ²=0.023 | 0.8804 |
|  | Yes | 51 (23.8%) | 25 (26.9%) | 76 (24.8%) |  |  |
| Hemoglobin (g/L) | Mean (SD) | 111 (22.8) | 108 (21.7) | 110 (22.5) | χ²=76.536 | 0.7325 |
|  | Median [Min, Max] | 114 [58.0, 168] | 108 [64.0, 154] | 113 [58.0, 168] | |  |
|  | Missing | 0 (0%) | 1 (1.1%) | 1 (0.3%) |  |  |
| WBC (×10⁹/L) | Mean (SD) | 8.27 (2.59) | 8.63 (3.19) | 8.38 (2.78) | χ²=167.25 | 0.2547 |
|  | Median [Min, Max] | 7.73 [4.62, 24.6] | 7.90 [5.40, 31.3] | 7.80 [4.62, 31.3] | |  |
| Neutrophils (×10⁹/L) | Mean (SD) | 5.10 (2.74) | 5.29 (3.21) | 5.16 (2.89) | χ²=156.04 | 0.5956 |
|  | Median [Min, Max] | 4.50 [0.600, 22.8] | 4.52 [1.80, 27.1] | 4.50 [0.600, 27.1] | |  |
| Platelets (×10⁹/L) | Mean (SD) | 193 (64.2) | 211 (70.6) | 199 (66.6) | χ²=175.51 | 0.4115 |
|  | Median [Min, Max] | 195 [37.0, 425] | 199 [73.0, 421] | 196 [37.0, 425] | |  |
| Albumin (g/L) | Mean (SD) | 35.7 (4.74) | 35.6 (3.59) | 35.7 (4.42) | χ²=126.89 | 0.5853 |
|  | Median [Min, Max] | 36.1 [3.00, 52.2] | 35.4 [23.8, 43.2] | 35.6 [3.00, 52.2] | |  |
| Lymphocytes (×10⁹/L) | Mean (SD) | 2.05 (0.518) | 2.13 (0.637) | 2.08 (0.557) | χ²=161.33 | 0.3684 |
|  | Median [Min, Max] | 1.95 [1.32, 5.32] | 1.98 [1.48, 6.66] | 1.96 [1.32, 6.66] | |  |
| PLR | Mean (SD) | 92.6 (31.3) | 99.6 (37.2) | 94.7 (33.3) | χ²=291.10 | 0.4708 |
|  | Median [Min, Max] | 88.5 [17.3, 199] | 95.7 [17.3, 208] | 89.4 [17.3, 208] | |  |
| NLR | Mean (SD) | 2.45 (1.00) | 2.40 (0.712) | 2.44 (0.922) | χ²=283.41 | 0.4988 |
|  | Median [Min, Max] | 2.38 [0.405, 9.10] | 2.37 [1.16, 4.52] | 2.38 [0.405, 9.10] | |  |
| SII | Mean (SD) | 462 (216) | 516 (248) | 479 (227) | χ²=296.67 | 0.4781 |
|  | Median [Min, Max] | 434 [64.1, 1290] | 473 [136, 1120] | 443 [64.1, 1290] | |  |
| Interval to surgery (days) | Mean (SD) | 44.4 (7.57) | 44.3 (7.62) | 44.4 (7.57) | χ²=37.807 | 0.1548 |
|  | Median [Min, Max] | 47.0 [27.0, 56.0] | 47.0 [27.0, 60.0] | 47.0 [27.0, 60.0] | |  |
| Surgical approach | Laparoscopic | 32 (15.0%) | 18 (19.4%) | 50 (16.3%) | χ²=0.014 | 0.9054 |
|  | Open | 182 (85.0%) | 75 (80.6%) | 257 (83.7%) | |  |
| Gastrectomy extent | Proximal | 41 (19.2%) | 21 (22.6%) | 62 (20.2%) | χ²=3.232 | 0.1987 |
|  | Total | 75 (35.0%) | 34 (36.6%) | 109 (35.5%) | |  |
|  | Distal | 98 (45.8%) | 38 (40.9%) | 136 (44.3%) | |  |
| Blood loss (mL) | Mean (SD) | 181 (94.9) | 191 (127) | 184 (105) | χ²=13.107 | 0.2177 |
|  | Median [Min, Max] | 200 [30.0, 1200] | 200 [30.0, 1200] | 200 [30.0, 1200] | |  |
| Operative time (min) | Mean (SD) | 147 (45.4) | 142 (37.0) | 146 (43.1) | χ²=42.19 | 0.3764 |
|  | Median [Min, Max] | 140 [70.0, 350] | 135 [64.0, 300] | 135 [64.0, 350] | |  |
| Postoperative complications (any) | No | 138 (64.5%) | 58 (62.4%) | 196 (63.8%) | χ²=0.051 | 0.8211 |
|  | Yes | 76 (35.5%) | 35 (37.6%) | 111 (36.2%) | |  |
| Vascular tumor thrombus | No | 96 (44.9%) | 45 (48.4%) | 141 (45.9%) | χ²≈0 | 1 |
|  | Yes | 118 (55.1%) | 48 (51.6%) | 166 (54.1%) | |  |
| Perineural invasion | No | 97 (45.3%) | 45 (48.4%) | 142 (46.3%) | χ²=0.015 | 0.9041 |
|  | Yes | 117 (54.7%) | 48 (51.6%) | 165 (53.7%) | |  |
| Tumor nodules | No | 153 (71.5%) | 64 (68.8%) | 217 (70.7%) | χ²=2.473 | 0.1158 |
|  | Yes | 61 (28.5%) | 29 (31.2%) | 90 (29.3%) |  |  |
| Lauren type | Unknown | 57 (26.6%) | 32 (34.4%) | 89 (29.0%) | χ²=2.236 | 0.5248 |
|  | Intestinal | 60 (28.0%) | 23 (24.7%) | 83 (27.0%) |  |  |
|  | Mixed | 62 (29.0%) | 21 (22.6%) | 83 (27.0%) |  |  |
|  | Diffuse | 35 (16.4%) | 17 (18.3%) | 52 (16.9%) |  |  |
| Signet-ring component | No | 169 (79.0%) | 63 (67.7%) | 232 (75.6%) | χ²≈0 | 1 |
|  | Yes | 45 (21.0%) | 30 (32.3%) | 75 (24.4%) |  |  |
| TRG (CAP) | C0 | 51 (23.8%) | 14 (15.1%) | 65 (21.2%) | χ²=3.827 | 0.2808 |
|  | C1 | 73 (34.1%) | 44 (47.3%) | 117 (38.1%) | |  |
|  | C2 | 50 (23.4%) | 19 (20.4%) | 69 (22.5%) |  |  |
|  | C3 | 40 (18.7%) | 16 (17.2%) | 56 (18.2%) |  |  |
| Histologic grade | Poor/Undifferentiated | 149 (69.6%) | 61 (65.6%) | 210 (68.4%) | χ²≈0 | 1 |
|  | Well/Moderate | 65 (30.4%) | 32 (34.4%) | 97 (31.6%) |  |  |
| Adjuvant chemotherapy | No | 134 (62.6%) | 55 (59.1%) | 189 (61.6%) | |  |
|  | Yes | 80 (37.4%) | 38 (40.9%) | 118 (38.4%) | |  |
| Resection margin (R) | R0 | 162 (75.7%) | 75 (80.6%) | 237 (77.2%) | χ²=0.641 | 0.4232 |
|  | R1 | 52 (24.3%) | 18 (19.4%) | 70 (22.8%) |  |  |
| CEA1 |  |  |  |  | χ²=0.644 | 0.4224 |
|  | <5 | 132 (61.7%) | 52 (55.9%) | 184 (59.9%) | |  |
|  | ≥5 | 82 (38.3%) | 41 (44.1%) | 123 (40.1%) | |  |
| CA1991 |  |  |  |  | χ²=1.964 | 0.1611 |
|  | <37 | 125 (58.4%) | 62 (66.7%) | 187 (60.9%) | |  |
|  | ≥37 | 89 (41.6%) | 31 (33.3%) | 120 (39.1%) | |  |
| CA1251 |  |  |  |  | χ²=0.173 | 0.678 |
|  | <35 | 122 (57.0%) | 52 (55.9%) | 174 (56.7%) | |  |
|  | ≥35 | 92 (43.0%) | 41 (44.1%) | 133 (43.3%) | |  |
| CA7241 |  |  |  |  |  |  |
|  | <7 | 124 (57.9%) | 54 (58.1%) | 178 (58.0%) | χ²=0.173 | 0.677 |
|  | ≥7 | 90 (42.1%) | 39 (41.9%) | 129 (42.0%) | |  |
| Supplementary Table S3 | | | | | | |

| Variable (comparison) | OR | 95% CI | P |
| --- | --- | --- | --- |
| Marital status (Single/Divorced vs Other) | 1.473363 | 0.790403–2.74645 | 0.223 |
| Intraoperative chemotherapy (Yes vs No) | 0.993338 | 0.684275–1.44199 | 0.972 |
| Hemoglobin (g/L, per unit) | 1.002754 | 0.995622–1.00994 | 0.45 |
| BMI (kg/m², per unit) | 1.022401 | 0.978816–1.06793 | 0.319 |
| Marital status (Married vs Other) | 1.745794 | 0.947898–3.21532 | 0.074 |
| NAC cycles (per cycle) | 1.001524 | 0.815934–1.22933 | 0.988 |
| Lauren type (Mixed vs Unknown) | 1.22375 | 0.790223–1.89512 | 0.366 |
| Lauren type (Intestinal vs Unknown) | 1.292595 | 0.830644–2.01145 | 0.255 |
| Histologic grade (Well/Moderate vs Poor/Undiff.) | 0.96669 | 0.677526–1.37927 | 0.852 |
| R status (R1 vs R0) | 2.657747 | 1.8751–3.76707 | <0.001 |
| Adjuvant chemotherapy (Yes vs No) | 0.316319 | 0.216006–0.463218 | <0.001 |
| Tumor location (Cardia vs Antrum) | 1.090809 | 0.739637–1.60871 | 0.661 |
| Tumor location (Corpus vs Antrum) | 1.896091 | 1.24247–2.89357 | 0.053 |
| Tumor location (Fundus vs Antrum) | 1.112642 | 0.4036–3.06732 | 0.837 |
| Tumor location (Pylorus vs Antrum) | 0.77665 | 0.311265–1.93785 | 0.588 |
| TRG (C1 vs C0) | 1.51993 | 0.975254–2.36881 | 0.064 |
| CEA (per unit) | 1.002376 | 0.990188–1.01471 | 0.704 |
| NAC adverse events (Yes vs No) | 0.69054 | 0.47011–1.01433 | 0.059 |
| Marital status (Unknown vs Other) | 1.936297 | 0.623426–6.01394 | 0.253 |
| Interval to surgery (days, per day) | 1.032888 | 1.01011–1.05618 | 0.004 |
| TRG (C2 vs C0) | 1.182596 | 0.724257–1.93099 | 0.503 |
| TRG (C3 vs C0) | 1.748222 | 1.05795–2.88887 | 0.029 |
| N stage (N1 vs N0) | 1.150863 | 0.653011–2.02828 | 0.627 |
| N stage (N2 vs N0) | 1.236334 | 0.76817–1.98982 | 0.382 |
| N stage (N3 vs N0) | 1.368596 | 0.840533–2.22841 | 0.207 |
| Lauren type (Diffuse vs Unknown) | 1.165251 | 0.697055–1.94792 | 0.56 |
| SII (per unit) | 1.00185 | 1.00112–1.00258 | <0.001 |
| NAC regimen (FOLFOX vs FLOT) | 2.030752 | 1.10734–3.72419 | 0.022 |
| NAC regimen (Other vs FLOT) | 1.051242 | 0.516721–2.1387 | 0.89 |
| NAC regimen (SOX vs FLOT) | 0.651483 | 0.382914–1.10842 | 0.114 |
| NAC regimen (XELOX vs FLOT) | 0.804262 | 0.454648–1.42272 | 0.454 |
| NAC regimen (Oral vs FLOT) | 1.417711 | 0.800945–2.50941 | 0.231 |
| Surgical approach (Open vs Laparoscopic) | 0.777464 | 0.498039–1.21366 | 0.268 |
| Tumor nodules (Yes vs No) | 0.943061 | 0.657579–1.35248 | 0.75 |
| T stage (T1 vs T0) | 0.017804 | 0.00190281–0.166584 | <0.001 |
| T stage (T2 vs T0) | 0.015419 | 0.00159772–0.148812 | <0.001 |
| T stage (T3 vs T0) | 0.017075 | 0.0018777–0.155274 | <0.001 |
| T stage (T4 vs T0) | 0.016491 | 0.00179735–0.151311 | <0.001 |
| Signet-ring component (Yes vs No) | 0.77782 | 0.515123–1.17448 | 0.232 |
| Age (per year) | 0.963462 | 0.927226–1.00111 | 0.057 |
| Albumin (g/L, per unit) | 1.038627 | 1.00304–1.07548 | 0.063 |
| Coronary artery disease (Yes vs No) | 0.92677 | 0.534259–1.60765 | 0.787 |
| Postoperative complications (Any vs None) | 0.929394 | 0.6594–1.30994 | 0.676 |
| Sex (Female vs Male) | 1.041297 | 0.731551–1.48219 | 0.822 |
| Gastrectomy extent (Distal vs Proximal) | 1.126665 | 0.705277–1.79982 | 0.618 |
| Blood loss (mL, per mL) | 0.993389 | 0.990256–0.996532 | 0.231 |
| Operative time (min, per min) | 1.003873 | 1.00018–1.00758 | 0.048 |
| Vascular tumor thrombus (Yes vs No) | 1.330172 | 0.956311–1.85019 | 0.09 |
| WBC (×10^9/L, per unit) | 1.049998 | 0.987865–1.11604 | 0.117 |
| Perineural invasion (Yes vs No) | 1.2908 | 0.930222–1.79115 | 0.127 |
| NLR (per unit) | 1.541535 | 1.33436–1.78087 | <0.001 |
| Platelets (×10^9/L, per unit) | 1.000033 | 0.997393–1.00268 | 0.98 |
| PLR (per unit) | 1.003121 | 0.997984–1.00828 | 0.234 |
| Diabetes (Yes vs No) | 1.296402 | 0.858527–1.9576 | 0.217 |
| Stroke history (Yes vs No) | 0.95077 | 0.61386–1.47259 | 0.821 |
| Lymphocytes (×10^9/L, per unit) | 1.276269 | 0.940779–1.7314 | 0.117 |
| Neutrophils (×10^9/L, per unit) | 1.111813 | 1.06595–1.15965 | <0.001 |
| Gastrectomy extent (Total vs Proximal) | 1.627721 | 1.01045–2.62209 | 0.045 |
| Hypertension (Yes vs No) | 0.974664 | 0.700072–1.35696 | 0.879 |
| CA1991>37 | 1.573243 | 1.135661202-2.179429 | 0.006431 |
| CA7241>7 | 1.556397 | 1.123501802-2.1560904 | 0.007808 |
| CEA1>5 | 1.486714 | 1.070355996-2.06503 | 0.018004 |
| CA1251>35 | 1.217146 | 0.875591539-1.6919368 | 0.242253 |
| Supplementary Table S4 | | | |

| Variable | Overall χ² | df | P | Nonlinear χ² | df | P | Determination |
| --- | --- | --- | --- | --- | --- | --- | --- |
| Neutrophils | 21.64 | 3 | 0.0001 | 5.77 | 2 | 0.0558 | Borderline nonlinear |
| Platelets | 6.52 | 3 | 0.0888 | 5.84 | 2 | 0.054 | Borderline nonlinear |
| SII | 7.81 | 3 | 0.0502 | 1.52 | 2 | 0.4665 | Linear |
| NLR | 21.1 | 3 | 0.0001 | 0.73 | 2 | 0.6925 | Linear |
| CEA | 3.63 | 3 | 0.3047 | 3.55 | 2 | 0.1693 | Linear |
| Albumin | 9.32 | 3 | 0.0253 | 2.94 | 2 | 0.2302 | Linear |
| BMI | 6.51 | 3 | 0.0891 | 4.88 | 2 | 0.0872 | Linear |
| Age | 6.69 | 3 | 0.0824 | 2.47 | 2 | 0.2913 | Linear |
| WBC | 4.35 | 3 | 0.2263 | 4.2 | 2 | 0.1222 | Linear |
| Lymphocytes | 4.35 | 3 | 0.2263 | 4.2 | 2 | 0.1222 | Linear |
| PLR | 1.22 | 3 | 0.7491 | 1.01 | 2 | 0.6039 | Linear |
| Hemoglobin | 0.98 | 3 | 0.8072 | 0.49 | 2 | 0.7833 | Linear |
| Interval to Surgery | 7.2 | 3 | 0.0657 | 1.3 | 2 | 0.5219 | Linear |
| Supplementary Table S5 | | | | | | | |
